# Screening of Eurasian Tundra Reindeer for Viral Sequences by Next-Generation Sequencing

**DOI:** 10.3390/ijerph18126561

**Published:** 2021-06-18

**Authors:** Javier Sánchez Romano, Anna Omazic, Mikael Leijon, Åsa Hagström, Morten Tryland, Juha Kantanen, Tiina Reilas, Ulrika Rockström, Valery Fedorov, Ann Albihn

**Affiliations:** 1 Arctic Infection Biology, Department of Arctic and Marine Biology, UiT The Arctic University of Norway, N-9019 Tromsø, Norway; morten.tryland@uit.no; 2 Vascular Biology Research Group, Department of Medical Biology, UiT The Arctic University of Norway, N-9019 Tromsø, Norway; 3 Department of Chemistry, Environment and Feed Hygiene, National Veterinary Institute, SE-751 89 Uppsala, Sweden; anna.omazic@sva.se (A.O.); ann.albihn@sva.se (A.A.); 4 Department of Microbiology, National Veterinary Institute, SE-751 89 Uppsala, Sweden; mikael.leijon@sva.se (M.L.); asa.hagstrom@sva.se (Å.H.); 5 Natural Resources Institute Finland (Luke), FI-31600 Jokioinen, Finland; juha.kantanen@luke.fi (J.K.); tiina.reilas@luke.fi (T.R.); 6 Farm and Animal Health, SE-753 23 Uppsala, Sweden; ulrika.rockstrom@gardochdjurhalsan.se; 7 Yakut Scientific Research Institute of Agriculture, 677001 Yakutsk, Russia; vfedorov_09@mail.ru

**Keywords:** *Rangifer tarandus*, NGS, virus screening, orthobunyavirus, arenavirus, flavivirus, herpesvirus, picornavirus

## Abstract

Reindeer husbandry is essential for the livelihood and culture of indigenous people in the Arctic. Parts of the herding areas are also used as pastures for farm animals, facilitating potential transmission of viruses between species. Following the Covid-19 pandemic, viruses circulating in the wild are receiving increased attention, since they might pose a potential threat to human health. Climate change will influence the prevalence of infectious diseases of both humans and animals. The aim of this study was to detect known and previously unknown viruses in Eurasian tundra reindeer. In total, 623 nasal and 477 rectal swab samples were collected from reindeer herds in Fennoscandia, Iceland, and Eastern Russia during 2016–2019. Next-generation sequencing analysis and BLAST-homology searches indicated the presence of viruses of domesticated and wild animals, such as bovine viral diarrhea virus, bovine papillomavirus, alcephaline herpesvirus 1 and 2, deer mastadenovirus B, bovine rotavirus, and roe deer picobirnavirus. Several viral species previously found in reindeer and some novel species were detected, although the clinical relevance of these viruses in reindeer is largely unknown. These results indicate that it should be possible to find emerging viruses of relevance for both human and animal health using reindeer as a sentinel species.

## 1. Introduction

Climate change and anthropogenic activities (e.g., altered land use, agricultural practices, changes in human populations) are major drivers of the emergence and re-emergence of infectious diseases [1]. Climate change is predicted to have a greater impact in Arctic and sub-Arctic regions than in other parts of the world [2,3]. The threat from new and/or emerging infectious diseases may play a critical role for the survival of reindeer herding now and in the future. Free-ranging reindeer have numerous opportunities to exchange microorganisms with wildlife animals, but they also have regular contacts with humans. Thus, reindeer may be regarded as a sentinel species for potential pathogen microorganisms circulating in natural ecosystems, which may be relevant for livestock and human health. Better knowledge of circulating viruses is also important to avoid and understand emerging infectious diseases and pandemics, and also a central part of the One Health concept concerning zoonotic infectious diseases circulating in the wild.

Zoonoses are of special importance in the context of climate change. It has been estimated that more than 70% of current human infections are zoonoses [4]. Thus, both animal and human health will most likely be affected by changes in the distribution and virulence of zoonotic pathogens caused by climate change. It is likely that only a small proportion of the viruses circulating in nature have been detected and investigated. Improved knowledge within this research area is thus important for public health, as exemplified during the outbreak and course of the Covid-19 pandemic.

Reindeer husbandry is of great importance in northern Fennoscandia (Finland, Norway, Sweden) and in the Russian Federation, both for livelihoods and for cultural values. In wintertime, i.e., after slaughter and before calving, there are around 600,000 reindeer in Fennoscandia and 2.5 million in the Russian Federation. There are populations of wild Eurasian tundra reindeer in Iceland and Norway, and some smaller populations of wild forest reindeer (*R. t. fennicus*) in Finland and western Russia. The wild reindeer population in Iceland originates from 35 semi-domesticated reindeer imported from Finnmark, Norway, in 1787 [5]. At present, the Icelandic summer population consists of approximately 6500 animals, which are kept at low density by controlled hunting.

Under current reindeer herding regimes, the number of diseases and clinical cases detected are restricted under normal conditions. Semi-domesticated reindeer are free-ranging for most of the year, utilizing remote forest and mountain pastures, usually with little close contact and handling by people. Thus, reduced reproductive success or production, disease cases in individual animals and even small disease outbreaks may occur unobserved and veterinary attention and clinical investigations are not common, in contrast to the situation for livestock.

For cervids, including semi-domesticated reindeer, the most apparent impact of climate change may be increased frequency of difficult grazing conditions in wintertime [6]. The predicted more frequent rain-on-snow events will create multiple layers of hard ice, making lichen and other winter forage unavailable for reindeer, and causing starvation and emaciation. Future loss and fragmentation of pastures and habitats due to various human activities (e.g., exploitation in the form of increased wind power, forestry, and mining) and high predator pressure will make it difficult for animals and reindeer herders to mitigate the effects of climate change [7].

Infectious diseases directly or indirectly associated with climate change may become an increasing threat. When a new infectious disease is introduced to an immunologically naïve population, the effects may be serious. In the Fennoscandian countries and the Russian Federation, herding systems and levels of pastoralism vary, and the occurrence and epidemiology of certain diseases can also be expected to vary. When weather extremes hinder the ability of reindeer to smell forage under the ice and reach it through digging, to avoid starvation reindeer are fed supplementary fodder in the field or as full maintenance in enclosures. This mitigation strategy saves reindeer lives, but also leads to stress, increased animal density, challenging hygiene conditions, and sometimes lack of clean snow or water for drinking, all of which increase the risk of infectious disease transmission. Thus, opportunistic infections might become a more frequent threat. Infectious diseases of the mucosa of the eyes and mouth are increasingly being observed [8,9]. Arctic wildlife and indigenous peoples’ health are especially at risk due to their dependence on subsistence food resources and the fact that climate change will have a greater impact in the area [10]. Therefore, a transdisciplinary One Health approach in northern regions is a must, i.e., better management of human health, animal health and ecosystem health of this and other remote regions, combining traditional and scientific experience and knowledge.

**Table 1:** Virus infections of known or potential clinical relevance identified to circulate in Eurasian tundra reindeer (*Rangifer tarandus tarandus*).

Virus	Information	References
*Flaviviridae*	Serological studies have reported pestivirus antibodies in reindeer from Finland, Norway, Sweden, and Iceland, as well as in caribou from Canada. The clinical relevance of pestivirus infections in reindeer is unknown. It may, however, be reasonable to assume that also reindeer may be persistently infected, with abortion, stillbirth, and the birth of persistently virus shedding offspring (i.e., persistently infected animals), as seen for many other host species. West Nile virus has also been demonstrated to infect reindeer, causing clinical disease.	[11,12,13,14,15]
*Herpesviridae* *Alphaherpesvirinae*	Cervid herpesvirus 2 (CvHV2) is enzootic in the Fennoscandian reindeer populations and antibodies against alphaherpesvirus have also been detected in caribou in Alaska (USA) and Canada. CvHV2 has been shown to act as the primary cause of infectious keratoconjunctivitis in reindeer during outbreaks and after experimental ocular inoculation, although many types of bacteria may contribute to the disease. CvHV2 may also cause respiratory infections in reindeer, and possibly abortion and weak-borne calves.	[16,17,18,19,20,21,22]
*Herpesviridae* *Gammaherpesvirinae* Genus *Macavirus*	The subfamily *Gammaherpesvirinae* contains several closely related virus species that are associated with malignant catarrhal fever (MCF). Sheep and goats are healthy carriers of ovine herpesvirus 2 and caprine herpesvirus 2, respectively, but may transmit the virus to susceptible domestic and wild ruminants. One clinical case of MCF in reindeer has been reported. The recorded symptoms were hair loss and thickening of the skin, with crusts in the axillary region, distal parts of the feet, and on the muzzle. Further, the animal had swollen eyelids, opaque cornea and fibrinopurulent eye discharge.	[23,24,25]
*Papillomaviridae*	Papillomaviruses cause mostly benign processes in the skin (papillomas, fibropapillomas or warts) or mucous membranes (condylomas) in many animal species, including reindeer. The clinical outcome may be serious for the individual. Papilloma viruses are considered species-specific, but several virus species may circulate in the same host species. The prevalence of papilloma viruses in reindeer is scarce. Generalized papillomatosis has been reported, affecting the skin in coalescing warts all over the body.	[25,26,27]
*Poxviridae* Genus *Parapoxvirus*	Orf virus (ORFV) and pseudocowpoxvirus (PCPV) have small ruminants and cattle as their main reservoirs. ORFV cause contagious ecthyma in and around the mouth in sheep and goats, and a similar disease has been reported in reindeer in Sweden, Finland, and Norway. Early outbreaks in Finland were caused by ORFV, whereas later outbreaks, from 1999–2000, have been associated with PCPV, with a milder clinical appearance as compared to ORFV.	[25,28,29,30,31]

Another example of a new threat is expansion of the geographical distribution of arthropod vectors and host animals, such as roe deer and badgers, due to climate change [32,33].

Some virus infections of known or potential clinical relevance are known to circulate in reindeer (Table 1). Among other relevant viruses, exposure of reindeer or caribou (wild, semi-domesticated, or captive) has been indicated for rabies virus (Canada, Svalbard, Russia), parainfluenzavirus 3 (PIV3) (Sweden), polyomavirus (Alaska), West Nile virus (USA), bluetongue virus (Germany), Schmallenberg virus (Germany), and foot-and-mouth disease virus (Russia) [25,34,35,36].

Ongoing climate change and other drivers affecting ecosystems may influence the type and nature of virus infections directly, or by impacting herding strategies and management. For most virus infections of relevance for reindeer, the transmission potential between wildlife, domesticated animals, and reindeer is not known. The aim of the present study was thus to detect potential virus infections circulating in reindeer populations in northern Fennoscandia, Iceland, and Eastern Russia (Yakutia). Better knowledge of the viruses circulating among reindeer will make it possible to predict health and disease challenges in the vulnerable reindeer herding industry, and to track changes due to increased anthropogenic encroachment and climate change over time.

## 2. Materials and Methods 

### 2.1. Ethical Statement

In Finland and Sweden, samples were obtained from slaughtered animals. In Norway, sampling was conducted in a general health surveillance of the herds when animals were gathered and handled for other herding purposes, and the study was not classified as an animal experiment. Animal handling procedures and sample collection were performed in accordance with regulations set by the Russian Authorization Board (FS/U.VN-03/163733/07.04.2016). In Iceland, opportunistic sampling from dead animals was perform during the hunting season, with appropriate permits from the Icelandic authorities.

### 2.2. Sample Collection

**Figure 1:**
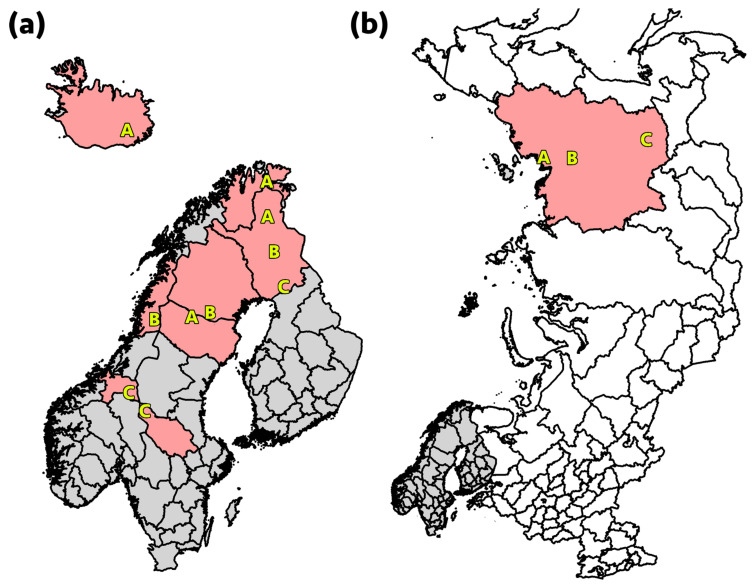
(**a**) In Finland, Norway, and Sweden, samples were collected from three geographical locations, denoted A, B, and C, reflecting different pasture and herding conditions. The sampling region in Iceland is also displayed (A). (**b**) The sampling regions in Ust-Yansky, northern Yakutia (A), Eveno-Bytantay, north-central Yakutia (B), and Aldan, southern Yakutia (C), Russia.

In total, 623 nasal and 477 rectal swab samples from Eurasian tundra reindeer (*Rangifer t. tarandus*) herds in Iceland, Finland, Norway, and Sweden, and the Republic of Sakha, Yakutia, Russia, were included in the study (Table 2 and Table 3, Figure 1). During the first year of sampling in the Nordic countries, eNAT swabs (Copan Italia, Brescia, Italy) were used, while for the remaining sampling UTM swabs (Copan Italia, Brescia, Italy) were used. The sampling performed in Yakutia was conducted with eNAT swabs (Copan Italia, Brescia, Italy) in 2017 and Amies Agar Gel with Charcoal Transport Swabs (JSHD Medical, Yancheng, China) in 2019. In Iceland, wild reindeer shot during the regular hunt were sampled. The reindeer sampled in the other countries were semi-domesticated. The samples from Finland and Sweden were obtained from slaughtered reindeer, whereas the samples from Norway were collected from live animals in corrals. Samples from Finland, Norway, and Sweden represented three geographical locations (Regions A, B, and C), reflecting different pasture and herding conditions. In Russia, sampling was performed during slaughter at two sampling locations (Regions A and B) in northern Yakutia (Figure 1b), while samples were obtained from live animals at one site (Region C) in southern Yakutia. Calves (≤1 year old) and adult animals (>1 year old) were both sampled, except in Iceland where only two calves were available due to a special permit in 2017. The samples were collected in two consecutive years at each site, during the period November 2016 to September 2018, in all countries except Russia, where sampling was performed at one site (Ust-Yansky, northern Yakutia) in December 2017 and at two sites (Eveno-Bytantay, north-central Yakutia, and Aldan, southern Yakutia) in November 2019. All animals sampled at slaughter were examined *ante mortem* by an official veterinarian and classified as healthy. The reindeer sampled in Iceland and Russia were all considered healthy by the hunters, slaughterers, or an official veterinarian, and were intended for human consumption.

### 2.3. Nucleic Acid Extraction

Before nucleic acid extraction, 1200 mL of swab collection buffer from each sample were initially filtered through a 0.45 µm filter to remove particles of bacterium size and larger. However, this filtration step was eventually excluded, since most samples were sufficiently clean and did not contain much debris. For swabs in eNAT buffer, five samples were pooled and 550 µL from the pool were used to extract nucleic acids with a magnetic bead-based kit (Viral NA Extraction Kit, Diasorin, Ireland) in an Arrow extraction robot (NorDiag, Oslo, Norway). For swabs in UTM buffer, buffer from each sample was mixed with 10xTURBO DNase Buffer (Kit TURBO DNase; Invitrogen, Carlsbad, CA, USA) to obtain a 1xTURBO DNase Buffer concentration, before pooling five samples per pool. Each UTM-buffer pool was treated with 2 U/µL TURBO DNase (Invitrogen, Carlsbad, CA, USA) to a concentration of 0.2 U/µL and 2.8 µL 40 U/µL of RNase One (Invitrogen, Carlsbad, CA, USA) at 37 °C for 30 min, to degrade unprotected nucleic acids. Then 250 µL was extracted from the pool with a magnetic bead-based kit (Viral NA Extraction Kit, Diasorin, Ireland) in an Arrow extraction robot (NorDiag, Oslo, Norway). Extracted RNA in the eluted total NA was converted to cDNA using random hexamers or the FR20RV-6N primer [37] with the SuperScript IV first-strand synthesis kit (Invitrogen). Double-stranded DNA was obtained by incubation of cDNA with Klenow Fragment DNA polymerase (New England Biolabs, Ipswich, MA, USA) at 37 °C for 1 h. The Klenow enzyme was then inactivated at 75 °C for 10 min. When the tagged primer was used for cDNA synthesis, random amplification of the tagged cDNA was performed using the FR20RV primer [37] under the following conditions: 10 min at 95 °C, followed by 40 cycles of 30 s at 95 °C, 30 s at 58 °C, and 90 s at 72 °C. The reaction was ended with an extra elongation step at 72 °C for 10 min. The PCR reaction contained 1x PCR buffer, 2.5 mM MgCl2, 2.5 mM dNTPs, 0.4 mM primer, and 1.25 U AmpliTaq Gold DNA polymerase (Applied Biosystems, Foster City, CA, USA). Some sample pools were run in triplicate with FR20RV primer, and then the products were pooled before purification by QIAquick PCR purification kit (Qiagen, Hilden, Germany) according to the manufacturer’s protocol [38]. The amplified DNA fragments were further treated with EcoRV (New England Biolabs) to remove the amplification primers and purified by QIAquick PCR purification kit (Qiagen, Hilden, Germany). Concentration was measured with a Qubit fluorometer using Qubit dsDNA HS (High Sensitivity) Assay Kit (Invitrogen, Carlsbad, CA, USA), and an 0.2 ng/µL aliquot was prepared for each sample.

### 2.4. NGS Library Preparation and Sequencing

Nextera XT DNA Library Preparation Kit (Illumina Inc., San Diego, CA, USA) was used to fragment the input DNA and tag the DNA from each sample with a pair of unique index primers by a 12-cycle PCR amplification. The libraries were purified with AMPure XP beads (Sigma-Aldrich, Milan, Italy), and Agilent High Sensitivity DNA Kit (Agilent Technologies, Waldbronn, Germany) was used to verify the length distribution of the fragments and for quantification of the libraries. Finally, an equimolar amount (preferably 4 nM, but when concentration was not high enough 2 nM was used) of each sample library DNA was pooled, denatured, and further diluted to a final concentration of 10 pM. Sequencing was performed on a MiSeq desktop sequencer using MiSeq 2 × 300 cycles reagent kit (v. 2) (Illumina, Inc.). Library preparation and sequencing was performed according to the manufacturer’s instructions.

### 2.5. Bioinformatics

The sequence reads were homology searched against the NCBI nt database using a Decypher server (TimeLogic^®^, Carlsbad, CA, USA). Before blasting, the sequence reads were quality checked and trimmed using HTStream [39]. Over 17 NGS runs the average read length after trimming was 216 nt and the average number of reads per run was 1,939,070. First, the trimmed reads were blasted against the VRL section of the NCBI nt database (i.e., the viral sequences) with a cut-off except value (e-value) of 10^−5^. A VRL blast database was created using the BLAST+ command line tools available from NCBI [40]. The reads that hit sequences in VRL within the limit of this e-value were collected with an in-house Python script and blasted against the whole nt database with the same e-value. The reads that again had the best hits (lowest e-value) to viral sequences were collected with an in-house Python script. This procedure reduced the computational burden on blasting against the large nt database by about 90%, since non-viral reads were filtered away against the much smaller VRL section.

## 3. Results

**Figure 2:**
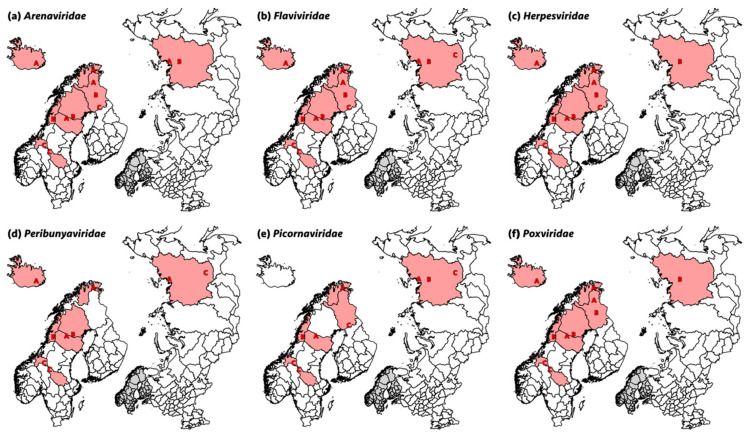
Maps showing the regions in Finland (A, B, C), Norway (A, B, C), Sweden (A, B, C), Iceland (A), and Russia (A, B, C) in which sequence read hits were detected for viruses from (**a**) the family *Arenaviridae*, (**b**) the family *Flaviviridae*, (**c**) the family *Herpesviridae*, (**d**) the family *Peribunyaviridae,* (**e**) the family *Picornaviridae*, and (**f**) the family *Poxviridae*.

Extracted nucleic acids from 477 rectal swabs pools and 623 nasal swab pools were processed for next-generation sequencing (NGS). Most swab sample pools produced sequences classified as viruses, but there was a tendency for pools from Finland and Russia to contain fewer or no viral sequences. A summary of results for the study regions with positive nasal and/or rectal swab pools for viruses from selected viral families can be found in Table 4 and Figure 2, while a complete overview of the NGS sequence reads with positive pools, read counts, and e-values (min) is provided in the Supplementary Materials. In this results section, the main findings for selected viral families are described in more detail. Sequence reads referred to as ‘virus sequence reads’ were classified by the BLASTn algorithm as most similar to that particular virus sequence in the current version of the nt database of NCBI GenBank. These classifications are also referred to as ‘hits’, meaning the best sequence hits using the BLASTn algorithm.

### 3.1. Arenaviridae

Overall, the most abundant sequence reads, both in number of pools and in geographical distribution (Figure 2a), were reads similar to various viruses from the family *Arenaviridae*, i.e., Lassa mammarenavirus (104 pools in the range 2–242), Guanarito mammarenavirus (85 pools in the range 2–866) and Luna mammarenavirus (three pools with one sequence read each). The range of sequence reads was 1–860 in the different pools. Sequence reads classified as *Arenaviridae* were detected in all countries and sampling years except for Finland (Region B) and Russia (Region C) in sampling 2. 

### 3.2. Flaviviridae

*Flaviviridae* sequence reads were very common and fell in the range 2–47 per pool when present. Sequence reads mainly belonged to two species, dengue virus 1 (14 pools in the range 2–11) and Iguape virus (at least 49 pools in the range 2–47), and both were present in all countries. Bovine viral diarrhea virus (BVDV) hits were also common and were found in 23 pools distributed among all countries except Russia (Figure 2b), although with very few reads in the range 1–4. Specific hits against BVDV were detected in Sweden (six pools), Norway (10 pools), Finland (three pools), and Iceland (four pools). The only other flavivirus hits were a single West Nile virus read from Norway (Region A) and two classical swine fever virus (CSFV) reads from Russia (Region A).

### 3.3. Herpesviridae

*Herpesviridae* hits fell into three subfamilies (*Alpha-*, *Beta-* and *Gammaherpesvirinae*)*. Gammaherpesvirinae* read counts were the most common, with hits in at least 30 pools distributed among all countries (Figure 2c) and generally with a high number of reads (range 1–54,453), especially in nasal swabs from Region C in Norway (sampling 2, minimum total read count 186,432 hits). *Alphaherpesvirinae* reads were detected in at least 39 pools and in all countries, although with lower read counts than *Gammaherpesvirinae* (range 1–7). *Betaherpesvirinae* sequence reads were also detected in a total of 18 pools in all countries, with high read counts again in nasal swabs from Region C in Norway (sampling 2; range 2–276). Most hits were against ruminant herpesviruses (e.g., ovine herpesvirus 2 (16 pools in the range 1–54,453), bovine herpesvirus 6 (13 pools in the range 1–36,279), or alcephaline herpesvirus 1 (11 pools in the range 2–36,279) and 2 (14 pools in the range 1–36,279), including reindeer gammaherpesvirus and cervid herpesvirus 3, with two sequence read hits in one pool each. 

### 3.4. Papillomaviridae

Papillomavirus hits were detected in all countries, and in both rectal and nasal swab pools. Read counts were low both in rectal (range 1–182) and in nasal swab pools (range 1–89). Interestingly, three hits against the reindeer papillomavirus were detected in one nasal swab pool in Iceland and one in Norway. Several hits against other ruminant papillomaviruses (e.g., bovine papillomaviruses or cervus elaphus papillomaviruses) were detected in 26 pools in Sweden, Finland, and Russia.

### 3.5. Paramyxoviridae

*Paramyxoviridae* reads were found in pools from all countries, but usually with only a few reads and often most similar to human respirovirus 1, with two sequence reads detected in 10 different pools. Norway was an exception, as two nasal swabs pools contained in total 37 sequence reads most like human respirovirus 3 (15 sequence reads in one pool), bovine respirovirus 3 (18 sequence reads in two pools), and caprine respirovirus 3 (four sequence reads in two pools).

### 3.6. Parvoviridae

Most hits belonging to the *Parvoviridae* family were associated with the red-crowned crane parvovirus, with read counts in the range 2–109 detected in 48 pools, but several pools also contained reads that hit various viruses of the genus *Bocaparvovirus*. *Parvoviridae* sequence reads were detected in 31 rectal swab pools (read count range 2–109) and in 22 nasal swab pools (read count range 2–48) in all countries.

### 3.7. Peribunyaviridae

Pools from all countries except Finland contained reads assigned to an *Orthobunyavirus* species with read counts detected in 34 pools in the range 2–23 (Figure 2d). In addition, three Simbu virus reads were found in one pool from Sweden and one from Russia, and six Ngari virus reads were found in four pools from Iceland, Norway, and Sweden.

### 3.8. Picobirnaviridae

*Picobirnavirus* hits were widespread in the pools and were found in 72 pools from all countries, with read counts in the range 1–31. The most common host species of these hits were marmot (eleven pools in the range 1–6), humans and other primates (32 pools in the range 1–31), and dromedary (eleven pools in the range 2–6).

### 3.9. Picornaviridae

*Picornaviridae* sequences were relatively rare and belonged to a diverse set of viruses. Sequence reads were detected in rectal swab pools from Norway, Sweden, Finland, and Russia (Figure 2e), and were most prominently identified as viruses from the genus *Kobuvirus* (17 pools in the range 2–53). As exceptions, one sequence read for human rhinovirus A was detected in one rectal swab pool from Sweden, and one rectal swab pool from Finland and one from Russia showed 46 sequence read hits to hepatoviruses (e.g., human hepatovirus A or hedgehog and rodent hepatoviruses). The only nasal swab pools in which picornavirus sequences were detected were from Region B in Russia, with one pool with sequence reads matching bovine rhinitis A (38 reads) and B (nine reads) virus, as well as foot-and-mouth disease virus type A (FMDV; six reads), all members of the genus *Apthovirus*.

### 3.10. Poxviridae

Low numbers of sequence reads belonging to the family *Poxviridae* were found in 11 rectal swab pools from all countries and in 13 nasal swab pools from all countries except Russia (Figure 2f). Most sequence reads matched orf virus (ORFV, genus *Parapoxvirus*;17 pools in the range 1–4), but sequence reads matching ruminant poxviruses of other genera (e.g., cowpox virus (CPXV), goat poxvirus (GPV), and white-tail deer poxvirus) were also detected in six pools with sequence reads in the range 1–4.

### 3.11. Small Circular DNA Viruses

As found in many other studies of fecal microbiome [37,41], many reads from small circular DNA viruses were observed in the present study, with most sequences belonging to the families *Circoviridae* (e.g., CRESS virus), *Genomoviridae* (e.g., *Alces alces* faeces assoc. genomovirus) and *Smacoviridae* (e.g., ovine faeces assoc. smacovirus 1 and bovine faeces assoc. smacovirus) (Supplementary Materials). In particular, small circular DNA viruses were especially prevalent in Norwegian rectal swab pools, with read counts detected in at least 14 out of 21 pools, but similar read counts were also identified in rectal swab pools from Sweden, Finland, and Iceland, and in nasal swab pools from Sweden and Norway. The clinical significance of these types of viruses has not yet been established.

### 3.12. Other Viruses

A variety of other viruses were detected in NGS analysis (Supplementary Materials). *Adenoviridae* hits were present in four pools (range 2–129) from Finland, Norway, and Sweden, and mostly belonged to ruminant viruses such as bovine adenoviruses (three pools with sequence reads in the range 4–129) or deer mastadenovirus B (one pool with two sequence reads). Astroviruses are frequently found in stool samples from many mammals, and ruminant astroviruses (e.g., bovine, deer, or yak astroviruses) were detected in at least eight rectal swab pools from Finland, Norway, and Sweden (range 1–44). Ruminant calicivirus hits (e.g., bovine calicivirus) were also detected in two pools from Norway (range 2–38). Reads for bovine rotavirus A and other reoviruses (e.g., human rotavirus A) were detected in seven rectal swab pools from Norway and Sweden, but with low numbers of hits (range 1–12). Interestingly, *Reoviridae* sequences were also detected in one nasal swab pool from Sweden and two from Norway, with two sequence read hits matching bluetongue virus in one of the Norwegian pools. Hits for human polyomavirus 12 and other polyomaviruses were only detected in one nasal swab pool from Norway. A variety of unclassified viruses, such as statovirus and Hainan astro-like virus 2, were also detected.

## 4. Discussion

Next-generation sequencing screening of viral pathogens in domestic animals and wildlife is an important tool to identify exposure to certain pathogens and help understand the etiology of diseases, but also to prevent possible disease outbreaks and identify emerging viral diseases in previously unexposed populations. In this study, Eurasian tundra reindeer in Iceland, Fennoscandia, and Yakutia, Russia, were screened for the presence of viruses. The sample set collected is unique in terms of the number of animals per country and the number of countries and sampling sites, representing a wide geographical coverage and spanning two winter seasons. Semi-domesticated reindeer are only available for sampling during the few times they are gathered during the reindeer herding year. Thus, these are presumably healthy animals that are gathered for tagging, selection of slaughter animals, etc. Sick animals in such herds will either be taken care of (caught, treated, or euthanized) or maybe never identified (survive and get healthy again, or die, usually never found due to scavengers, sometimes killed by predators). Sampling reindeer during regular herding practices is carried out under field conditions, and contamination of the nostrils and rectum of the animals with environmental or human material can occur during this procedure. Therefore, some of the viral material identified during this study may have been introduced during the animal handling and/or sampling procedure (e.g., human respiroviruses, papillomaviruses or herpesviruses, or red-crane parvovirus). Whether the sequence reads represent environmental/human contamination or a reindeer-specific virus needs to be elucidated. However, even in the case of contamination, the presence of this viruses may as well happen without the direct involvement of the sampler, due to the direct handling of the animals by reindeer herders during gathering, marking, and slaughtering. Even though sampled animals were considered healthy upon examination, a large variety of nucleic acid sequences of viral origin were detected in nasal and rectal swab pools from all countries studied. Therefore, it is possible that apparently healthy semi-domesticated reindeer may have a role as a pathogen reservoir for both domestic animals and wildlife, but also contribute to the transmission by meat and milk consumption, contact, and so on, of zoonotic pathogens to humans (e.g., Hepatitis E virus or ORFV) [9,42,43].

The method employed to detect the presence of viral nucleic acid sequences was to compare the sequence reads with the NCBI GenBank nt database (using the BLASTn algorithm) and collect the cases where the sequence reads were most similar to a viral sequence deposited in GenBank (‘best hit’), irrespective of the host species of this viral sequence. This method enables a first screening of large amounts of data, but has several drawbacks. For example, if the virus sequenced is lacking in the database, some other distantly or closely related virus will be the best hit, or there will be no hit at all. This incompleteness of the database will limit the precision of virus discovery. Other sources of false classification are parts of the host genome or microbial nucleic acids that are absent from the database. In such cases, these unknown nucleic acid sequences present in the sample may end up as viral sequences. In addition, the large nt database is in part uncurated and may contain erroneous sequences, giving rise to false hits. Other issues are contamination of reagents with various genomic material and the difficulty in distinguishing between-sample leakage of reads and between-run carry-over contamination that may occur on the Illumina MiSeq platform used in the present work. With these limitations in mind, we chose to report all virus hits obtained from the sample pools, under the condition that at least two reads hit the same or taxonomically closely related viruses. Findings that only relate to viral sequences from host species taxonomically very distant from reindeer, or otherwise less likely to infect reindeer, should be considered highly uncertain. Furthermore, samples were pooled, due to limited available funding, representing dilution of viruses in each pool. However, all individual samples have been preserved, making it possible to explore interesting pools in future studies. 

In the present study, human viruses (e.g., Lassa mammarenavirus, dengue virus) and non-reindeer-specific ruminant viruses (e.g., ruminant gammaherpesviruses, bovine papillomavirus) may have been overrepresented in comparison with reindeer-specific viruses, due to the lack of reindeer-specific viral sequences in the NCBI database. Therefore, it can be assumed that several of the hits found belong to specific reindeer viruses or other viral species that can infect reindeer. In fact, there is reason to believe that the sequence reads hits for *Gammaherpesvirinae* indicate a host-specific reindeer virus (rangiferine gammaherpesvirus 1) previously identified in semi-domesticated and wild reindeer in Norway [44,45]. The same applies for papillomaviruses, which are in general host-specific, with one or several papillomavirus species associated with a single host but sharing homologue sequences in parts of their genome [26].

The climate in the Arctic and sub-Arctic region is changing faster than the global average [46]. General knowledge on climate change effects and adaptation strategies has increased significantly in recent years, but there is still a substantial information gap regarding the influence of climate change on infectious diseases. In a One Health perspective, zoonotic infections are a particular concern, and we need more knowledge of what is present in the wild environment. Both animal and human health will most likely be affected by changes in the distribution and virulence of zoonotic pathogens caused by climate change, but also by other anthropogenic drivers and new animal hosts. Further, a population of humans or animals not previously exposed to a particular disease is immunologically naïve, so an outbreak of that disease in a new area (i.e., high-latitude regions) will likely have more severe effects.

The changing climate will give opportunities for climate-sensitive infectious diseases to establish or occur sporadically in new areas [47]. Vector-borne diseases are a particular concern in this regard. Arthropod vectors (e.g., ticks, mosquitoes, and midges) and reservoir animals (e.g., rodents, birds, and wild ungulates) for infectious diseases might both extend their distribution northwards as a result of changes in ecosystems associated with climate warming [48,49]. The rate of development, persistence, and multiplication of most arthropods and microorganisms is directly affected by microclimatic conditions, especially temperature. Warmer temperatures affecting activity and population dynamics of vectors may increase transmission of pathogens and result in spread to new environments. Warmer temperatures at high latitudes may also result in a longer vegetation period, making it easier for arthropod hosts to reproduce and thus develop denser populations [50].

Sequence reads indicating viruses from the family *Arenaviridae* were detected in reindeer from all countries studied. In general, most arenaviruses are only present in the southern hemisphere, with lymphocytic choriomeningitis virus being the only one described in Europe [51]. No arenavirus has yet been described in any *Rangifer* species. The widespread detection of sequence hits with high read counts against viruses from the family *Arenaviridae* raises the question of whether there is an unknown widespread arenavirus in reindeer. Alternative explanations are that the hits belong to one or more circulating viruses with similar genome sequences, or that the reads are homologs to the reindeer genome itself. Incidental integration of non-retrovirus RNA viruses, such as arenaviruses, has been described [52], and it is possible that such integration may have happened in the reindeer genome in the past, with the subsequent detected hits. In either case, further investigations to clarify this matter are necessary, since some arenaviruses are known to cause severe viral hemorrhagic fevers in humans through contact with infected rodents [53], and since the arenaviruses detected may be pathogenic to reindeer.

Blast hits for the family *Flaviviridae* were detected in reindeer from all countries studied. Most of the sequences matched dengue virus and Iguape virus (genus *Flavivirus*). However, it is highly unlikely that these viruses are circulating in Arctic reindeer populations. Another member of the genus *Flavivirus* with positive hits was West Nile virus (WNV), with a single sequence read hit from one nasal swab pool in Norway. Different wild mammals present in the Arctic are known to be flavivirus hosts [54], but flavivirus infections in reindeer have only been described for WNV [12]. Most known flaviviruses are arthropod-borne viruses, with mosquitoes and ticks as intermediate hosts. The arthropod-borne nature of WNV and other flaviviruses, and the fact that they can circulate, be introduced by, and maintained in migratory birds as reservoirs [55], make flaviviruses a risk to the Arctic reindeer population. In recent years, the mosquito *Culex modestus* Ficalbi 1889 has been identified as one of the main bridge vectors of WNV between birds and mammals, and it appears to have spread in northern and central Europe [56,57]. Climate change, diverse feeding habits, and increased vector competence may have made *Cx. modestus* more robust to high latitudes [58,59]. However, in Sweden, seropositivity for WNV has been detected only in nonresident birds, which is not considered indicative of local transmission [60].

Classical swine fever virus (CSFV, Pestivirus C) is closely related to BVDV and BDV, but only pigs and wild boars are considered natural reservoirs of this virus [61]. Experimental infection of several ruminants has been reported, but there is no evidence of natural infection of reindeer or other cervids under natural conditions. According to the World Organisation for Animal Health [62], the Nordic countries are officially CSFV-free, but the status in Russia is uncertain, with outbreaks reported in 2014. The two sequence reads against CSFV were detected in Russia (Region A), which may indicate circulation of CSFV in that area. However, the sequence reads could also belong to a different pestivirus species.

The remaining hits for *Flaviviridae* belonged to BVDV (pestivirus A and B; genus *Pestivirus*). Most cattle farms in the Nordic countries are currently considered BVDV-free, especially in the reindeer husbandry areas, after successful BVD eradication programs in the 1990s [63]. However, BVDV hits were detected in 23 pools, from Finland (three pools), Norway (10 pools), Sweden (six pools), and Iceland (four pools). To date, only one reindeer pestivirus has been isolated (pestivirus reindeer-1, V60-Krefeld) and sequenced [64]. The lack of genome sequences in the databases hampers identification of reindeer-specific pestivirus sequences by NGS. These findings, together with data from a previous serological screening [14], hint at the possibility that a reindeer-specific pestivirus, presumably closely related to BDV, is circulating among wild and semi-domesticated reindeer populations and may be responsible for these hits [15].

Several herpesviruses are known to infect and cause disease in Eurasian tundra reindeer (Table 1). One of the most common reindeer pathogens is the cervid herpesvirus 2 (CvHV2; subfamily *Alphaherpesvirinae*, genus *Varicellovirus*), which is enzootic in semi-domesticated reindeer, with seroprevalences reported to be ~50% [21,65,66]. Surprisingly, no hits against CvHV2 were detected in the present study. However, several hits against *Alphaherpesvirinae* were detected in 39 pools in all countries studied, suggesting that CvHV2 was in fact the virus generating the sequence hits. Once again, underrepresentation of a reindeer-specific virus may be the reason for the lack of hits if the sequences belong to highly conserved genes among herpesviruses, such as the UL24 gene or the glycoprotein B or H genes [67,68]. Sequence read hits for viruses from the subfamily *Gammaherpesvirinae* were common, with sequence reads in a total of 30 pools representing all countries. Most of the *Gammaherpesvirinae* viruses belonged to the malignant catarrhal fever virus group (MCFV; genus *Macavirus*), e.g., ovine herpesvirus 2 or alcephaline herpesvirus 1 and 2, and undetermined gammaherpesviruses. Read counts were especially high in Norway, with most hits generated from nasal swab pools from Region C in sampling 2. Those pools had a maximum sequence read count of 54,453 against ovine herpesvirus 2 (OvHV-2) and a minimum amount of 186,450 sequence hits for *Gammaherpesvirinae* in general. Betaherpesviruses are not often considered when discussing semi-domesticated reindeer health. This study detected *Betaherpesvirinae* sequences in all countries examined, including hits for cervid herpesvirus 3, a betaherpesvirus first identified in the eyes of semi-domesticated reindeer in Norway [27].

This study detected blast hits against an orthobunyavirus (family *Peribunyaviridae*) in all countries, with the exception of Finland. Sequence hits were in general low (2–4) except for Sweden, which had a maximum sequence read count of 23. Orthobunyaviruses have a wide geographic and host range, although individual viruses may be restricted to a small number of host species [69]. Adverse veterinary outcomes include fetal abnormalities and abortion storms among livestock (e.g., Schmallenberg virus; SBV). SBV, transmitted by biting midges (*Culicoides* spp.), first emerged in Europe in 2011 and in Sweden in late 2012. The virus then spread rapidly north beyond the Arctic Circle, occurring in high prevalence after the vector season in 2012 [70]. However, the virus has not been detected in Swedish domestic animals or circulating among wild cervids since the vector season in 2014 [71]. Even though SBV has not been detected in semi-domestic or wild reindeer in their natural range, the presence of seropositive reindeer in zoological parks in Germany demonstrates the susceptibility of reindeer to infection [40]. Northern Fennoscandia has a long vector-free winter season compared with ecosystems in central and southern Europe. Virus transmission and spread are possible at temperatures around 15 °C [72], and in northern Fennoscandia daily mean temperatures at this level are usually limited to May–August [73]. Virus persistence depends on the winter survival of adult midges, which must have access to an immunologically naïve ruminant population. If SBV is introduced to the reindeer population in Sweden or in one of the other countries studied, the effects may be serious. However, based on northern latitude climate conditions, it can be assumed that this region has an unfavorable climate for overwintering SBV vectors. In addition, midge activity and the reproductive season of Swedish wild cervids are seasonal and biological mismatches for the virus, which may explain why SBV has so far had little impact on Swedish wild ruminant health. These animals are thus highly unlikely to be reservoirs of this virus. Thus, the findings in the present study indicate that an unknown orthobunyavirus, different to SBV, may be circulating in the reindeer populations studied.

Papillomaviruses are considered species-specific, and to date only rangifer tarandus papillomavirus 1 (reindeer papillomavirus) has been isolated from semi-domesticated reindeer [26]. However, two other rangifer papillomaviruses have recently been characterized, in Norwegian reindeer [27] and Western Arctic caribou [36]. Papillomavirus sequence reads were detected in nasal and rectal swab pools in all countries in the present study. However, only one hit against the reindeer papillomavirus was detected, in a nasal swab pool from Iceland.

All sequence reads from the family *Paramyxoviridae* belonged to the genus *Respirovirus*. Human respirovirus 1 sequence reads were the most common among sequences from this genus and were detected mostly in nasal swab pools from all countries, with 15 sequence reads from human respirovirus 3 also detected in a nasal swab pool from Norway (Region C). Both viruses are considered human parainfluenza viruses, known pathogens of the respiratory tract which cause acute respiratory disease [74]. Bovine and caprine respirovirus 3 are also parainfluenza viruses which cause disease in ruminants, and sequence reads for these viruses were detected in two nasal swab pools from Norway (Region C). A previous serological screening for antibodies against bovine parainfluenza 3 reported 53% seroprevalence in Swedish reindeer [35]. All viruses in the *Respirovirus* genus seem to exhibit considerable genetic and antigenic similarity, and thus the presence of a reindeer-specific respirovirus cannot be discarded as a possibility.

Once again, overrepresentation bias towards other more common papillomaviruses or respiroviruses may have influenced the results of the sequence matches, with several other ruminant and human viruses in several swab pools instead of reindeer-specific viruses. On the other hand, one should not discard the possibility of a novel reindeer papillomavirus or respirovirus, or the possibility of human papillomaviruses and respiroviruses being present in the swab pools due to contamination during sampling or processing of the swabs.

While the majority of sequences belonging to the family *Parvoviridae* matched red-crowned crane parvovirus, hits against several bocaparvoviruses were also detected. Parvovirus infections can be associated with a variety of clinical signs, ranging from asymptomatic infections to severe disease, depending on the species [75]. Evidence of the presence of a caribou-specific parvovirus has been reported [36]. Whether the sequence reads represent environmental contamination or a reindeer-specific parvovirus needs to be further investigated. In several other projects, we also observed red-crowned crane parvovirus hits (unpublished data), so the validity of these should be regarded as highly uncertain.

*Picobirnavirus* is the only genus in the family *Picobirnaviridae*. Sequence reads from this genus were detected in nasal and rectal swabs from all countries studied. Several species of picobirnaviruses have been described as infecting mammals, but they have not been clearly linked to disease [76]. Only one species has so far been isolated from ruminants, roe deer picobirnavirus [76], which was detected in two rectal swab pools from Russia (Region C). However, most of the sequence read hits in this study were identified as marmot, human and other primates, and dromedary picobirnaviruses.

Most *Picornaviridae* sequences were detected in rectal swab pools and matched viruses of the genus *Kobuvirus* (e.g., Aichivirus A and B, or caprine and bovine kobuvirus). Kobuviruses are known to infect the gastrointestinal tract of several mammal species, causing gastroenteritis and diarrhea. Only three ruminant *Kobuvirus* species have so far been isolated, bovine (Aichivirus B1 and D), caprine (Aichivirus C2), and ovine (Aichivirus B3) kobuviruses [77], but kobuvirus RNA has also been detected in roe deer [78]. The widespread distribution of kobuviruses detected in rectal swab pools from reindeer in Fennoscandia and Yakutia, Russia, may indicate that at least one virus in this genus infects these reindeer populations. Additional studies are needed to determine whether this virus is a novel kobuvirus and to establish the epidemiological and clinical importance of kobuviruses in semi-domesticated and wild Eurasian tundra reindeer. One sequence read for human rhinovirus A (genus *Enterovirus*) and 46 sequences reads for hepatovirus A (e.g., human hepatitis A virus and other hepatoviruses) were also detected in rectal swab pools. Sequence reads matching viruses from the genus *Apthovirus* were only detected in one nasal swab pool, from Yakutia, Russia (Region C), with 47 sequence hits for bovine rhinitis virus A and B and six sequence reads for FMDV, a known and important pathogen which causes foot-and-mouth disease (FMD) in cattle and other domestic ruminants. FMD is a notifiable disease and is currently absent from the Nordic countries and most of the European Union, which has protocols in place to avoid the spread of FMDV. Russia is also mainly considered FMD-free, but outbreaks of FMD have recently been reported in far-east Russia [79]. Although FMD has been reported in reindeer and other wild and semi-domesticated ungulates, it apparently fails to establish in wildlife and it is most likely maintained in livestock, with sporadic spread to wild and semi-domestic ungulates [80,81].

*Poxviridae* sequences were detected in rectal swab pools from all countries studied here and in nasal swab pools from all countries except Russia. Most sequence reads matched ORFV, which is a member of the *Parapoxvirus* genus causing contagious ecthyma in small ruminants, reindeer, and many wildlife species, and a zoonotic infection [25]. ORFV-specific genome sequences have been detected by PCR in reindeer with no clinical signs of contagious ecthyma, indicating that the virus may circulate among reindeer without presenting as regular disease outbreaks [82]. In our experience, it is very common to observe a limited amount of ORFV reads in samples from various ungulates, including reindeer, using the NGS technology (unpublished observations). This may, in fact, indicate presence of the virus, since ORFV may have a broad host range among wild ungulates [83]. However, since the whole genome sequence of several *Parapoxvirus* species are available in GenBank, the matching NGS reads may also reflect similarities between certain immunomodulatory components of the virus and the host [84]. One example is the viral interleukin ortholog (*vIL-10*), which needs to have close similarity to the interleukin-10 of the host if the virus is to achieve effective replication [85]. Thus, the finding of poxvirus sequences in the present screening needs to be further substantiated on nucleotide sequence level.

New climatic conditions and landscape alterations have also contributed to the presence and altered distribution of other ungulates (e.g., roe deer or wild boar) [86,87] that can act as reservoirs of several viruses (e.g., CSFV, FMDV, ORFV, bluetongue virus, or SBV) that may be transmitted to semi-domesticated reindeer in the same areas [88,89]. At the same time, the detection of sequence reads belonging to some of those viruses in apparently healthy reindeer may indicate that after transmission to semi-domesticated reindeer, this species may have a role as reservoir in the subsequent transmission to other domestic and wild animals in the area, but also humans [9,40,41]. However, the possible role of semi-domesticated reindeer as a reservoir needs to be further investigated and cannot be inferred from the current data.

This screening of Eurasian tundra reindeer for viruses by NGS identified several viral families and species that can affect human and animal health in all countries and sampling sites studied. However, only a few of these virus families and species are recognized as being pathogenic for reindeer. Although the NGS screening method has limitations with regard to identifying pathogenicity and a potential causative role for a virus to cause a certain disease, it proved useful in suggesting potential pathogens present in Eurasian tundra reindeer as the host species. This first screening involved a significant number of reindeer samples, representing a broad geographic region and five countries. The results obtained should be further analyzed by addressing the gene sequences generated and conducting phylogenetic studies.

## 5. Conclusions

This screening of Eurasian tundra reindeer for viruses by NGS identified numerous viral families, including several species that can impair the health of reindeer, wildlife, livestock, and humans. A One Health perspective on further studies of these risks is vital. Climate change and other anthropogenic drivers will expand the future distribution of infectious diseases to new areas, ecosystems, and hosts.

This study showed that a large variety of virus species are circulating in the reindeer populations in all five countries studied. Only a few of these virus species are currently recognized as being pathogenic for reindeer. Some of the hits identified may belong to reindeer-specific pathogens that are underrepresented in GenBank (e.g., CvHV2, reindeer gammaherpesvirus, and parvovirus), thus generating ‘best hits’ with similar viruses associated with other hosts. However, several hits may belong to novel reindeer viruses (e.g., kobuvirus, picobirnavirus, arenavirus) with unknown impacts on reindeer populations. These novel viruses could represent a potential health risk for reindeer, other animal species, and humans, so further studies are needed to identify their pathogenic potential.

## Figures and Tables

**Table 2:** Details of the 623 nasal swabs obtained from 484 Eurasian tundra reindeer (*Rangifer t. tarandus*), including calves (≤1 year) and adult animals (>1 year), in Finland, Norway, Sweden, Iceland, and Russia. Swabs were taken from three geographically separate herds in each country except for Iceland, where the wild reindeer population was sampled.

Sampling Site	Sampling 1				Sampling 2			
	Time of Sampling	Total no. of Reindeer	No. of Calves	No. of Adults	Time of Sampling	Total no. of Reindeer	No. of Calves	No. of Adults
Finland, A	December 2016	19 ^1^	10	9	November 2017	22	13	9
Finland, B	January 2017	20 ^1^	14	6	October 2017	20	10	10
Finland, C	February 2017	21 ^1^	10	11	October 2017	20	10	10
Norway, A	November 2016	20 ^1^	10	10	November 2017	20	11	9
Norway, B	January 2017	20 ^1^	10	10	April 2018	21	11	10
Norway, C	January 2017	20 ^1^	10	10	January 2018	20	10	10
Sweden, A	December 2016	20	10	10	December 2017	20	10	10
Sweden, B	November 2016	33 ^2^	14	9	December 2017	20	10	10
Sweden, C	November 2016	19 ^1^	9	10	November 2017	20	10	10
Iceland	August 2017	25	2	23	September 2018	24	0	24
Russia, A	December 2017	20	4	16	n/a	n/a	n/a	n/a
Russia, B	n/a	n/a	n/a	n/a	Nov 2019	20	0	20
Russia, C	n/a	n/a	n/a	n/a	Nov 2019	20	0	20
Total		237	103	124		247	95	152

^1^ During the first-year sampling in Norway, Sweden (only site C) and Finland, swabs from both left and right nostril were collected. ^2^ Ten of the sampled animals were of unknown age.

**Table 3:** Details of the 477 rectal swabs Eurasian tundra reindeer (Rangifer t. tarandus), including calves (≤1 year) and adult animals (>1 year), in Finland, Norway, Sweden, Iceland, and Russia. Sampling was performed during two consecutive years. Swabs were taken from three geographically separate herds in each country except for Iceland, where the wild reindeer population was sampled.

Sampling Site	Sampling 1				Sampling 2			
	Time of Sampling	Total no. of Reindeer	No. of Calves	No. of Adults	Time of Sampling	Total no. of Reindeer	No. of Calves	No. of Adults
Finland, A	December 2016	19	10	9	November 2017	21	13	8
Finland, B	January 2017	21	14	7	October 2017	20	10	10
Finland, C	February 2017	21	10	11	October 2017	20	10	10
Norway, A	November 2016	20	10	10	November 2017	19	11	8
Norway, B	January 2017	20	10	10	April 2018	20	10	10
Norway, C	January 2017	20	10	10	January 2018	19	9	10
Sweden, A	December 2016	20	10	10	December 2017	20	10	10
Sweden, B	November 2016	30 ^1^	13	7	December 2017	20	10	10
Sweden, C	November 2016	20	10	10	November 2017	20	10	10
Iceland	August 2017	25	1	24	September 2018	22	0	22
Russia, A	December 2017	20	4	16	n/a	n/a	n/a	n/a
Russia, B	n/a	n/a	n/a	n/a	Nov 2019	20	0	20
Russia, C	n/a	n/a	n/a	n/a	Nov 2019	20	0	20
Total		236	102	124		241	93	148

^1^ Ten of the sampled animals were of unknown age.

**Table 4:** Summary of regions with positive nasal and/or rectal swab pools (X) for viruses from selected viral families. Nasal and rectal swabs were collected from three different semi-domesticated reindeer herds in Finland, Norway, and Sweden (regions A, B, and C) and from wild reindeer in Iceland (region A) in in two consecutive sampling years (samplings 1 and 2). Nasal and rectal swabs were collected and pooled from one semi-domesticated reindeer herd in Yakutia, Russia, in 2016 (region A) and two different herds in 2019 (regions B and C).

Virus Family	Sweden						Norway						Finland						Iceland		Russia		
	Sampling 1			Sampling 2			Sampling 1			Sampling 2			Sampling 1			Sampling 2			Sampling 1	Sampling 2	Sampling 1	Sampling 2	
	A	B	C	A	B	C	A	B	C	A	B	C	A	B	C	A	B	C	A	A	A	B	C
*Adenoviridae*		X							X				X					X					
*Arenaviridae*	X	X	X	X	X	X	X	X	X	X	X	X	X	X	X	X		X	X	X	X	X	
*Astroviridae*			X				X	X							X								
*Caliciviridae*								X															
*Flaviviridae*	X	X	X	X	X	X	X	X	X		X	X	X	X	X		X	X	X	X	X	X	X
*Herpesviridae*	X		X	X	X	X	X	X	X	X	X	X	X	X	X	X	X	X	X	X		X	
*Papillomaviridae*	X				X		X	X	X		X	X			X			X	X		X		
*Paramyxoviridae*	X	X	X	X		X	X	X	X										X				X
*Parvoviridae*	X	X	X	X	X	X				X	X	X	X	X	X			X		X	X		X
*Peribunyaviridae*	X	X	X		X	X	X	X	X										X	X	X		X
*Picobirnaviridae*						X		X		X	X	X		X	X	X	X	X		X	X	X	X
*Picornaviridae*	X		X					X	X	X		X		X							X	X	X
*Poxviridae*	X	X	X	X	X	X	X	X	X			X	X	X					X	X		X	
Small circular DNA viruses			X	X	X	X	X	X	X	X	X	X		X	X		X	X	X	X			

## Data Availability

The data presented in this study is available as Supplementary Material and can be accessed online at https://www.mdpi.com/article/10.3390/ijerph18126561/s1.

## Supplementary Materials

The following tables are available online at https://www.mdpi.com/ article/10.3390/ijerph18126561/s1. Complete dataset with viral Blastn hits from rectal and nasal swabs collected from semi-domesticated and wild Eurasian tundra reindeer (*Rangifer tarandus tarandus*) in Sweden, Norway, Finland, Iceland and Russia in different geographical locations and during two separate sampling periods.
