# Supplementary material for: Screening of Eurasian Tundra Reindeer for Viral Sequences by Next-Generation Sequencing
Source: Int J Environ Res Public Health. 2021 Jun 18;18(12):6561. doi: 10.3390/ijerph18126561 (PMC8296488; doi:10.3390/ijerph18126561)
Supplement: Supplementary file 1 [file ijerph-18-06561-s001.zip › ijerph-1186023-supplementary.pdf]

Supplementary Table S1. Viral Blastn hits from rectal swabs collected from semi-domesticated Eurasian tundra reindeer in Sweden during late autumn 2016. Reindeer were sampled in herds from three regions (A, B, C).

**Virus**	**Region A 08-11-2016**			**Region B 06-12-2016**			**Region C 01-11-2016**		
	**Pools**	**e-value (min)**	**Read count**	**Pools**	**e-value (min)**	**Read count**	**Pools**	**e-value (min)**	**Read count**
Arenaviridae	*6/6*			3/3			4/4		
Guanarito mammarenavirus	6/6	4E-54–2E-50	100–866	3/3	2E-52–7E-51	105–398	4/4	1E-50–4E-49	13–180
Lassa mammarenavirus	6/6	6E-80–3E-59	15–109	3/3	1E-73–2E-59	16–36	3/4	8E-69–1E-61	5–75
Astroviridae									
Bovine astrovirus							2/4	1E-59–5E-52	2
Water buffalo astrovirus							1/4	1,00E-48	1
Flaviviridae				1/3			1/4		
Iguape virus	4/6	1E-56–2E-43	3–10	1/3	2,00E-32	3	1/4	3,00E-27	3
Dengue virus	1/6	3,00E-10	2	1/3	4,00E-09	2			
Bovine viral diarrhea virus	1/6	5,00E-15	1						
Herpesviridae									
Alphaherpesviridae	1/6								
Felid herpesvirus 1							1/4	2,00E-06	1
Mule deer alphaherpesvirus	1/6	7,00E-12							
Suid herpesvirus 1							2/4	6E-13-1E-11	2
Gammaherpesviridae									
Human herpesvirus 4	2/6	9,00E-07							
Ovine/caprine herpesvirus 2	2/6	5E-15–9E-6							
Papillomaviridae									
Bovine papillomavirus	2/6	2E-36–7E-15	2–4						
Human papillomavirus type 115	1/6	4,00E-28	2						
Pudu puda papillomavirus type 1	1/6	5,00E-78	1						
Paramyxoviridae	*1/6*			1/3			1/4		
Human Respirovirus 1	1/6	9,00E-24	2	1/3	2,00E-26	2	1/4	2,00E-48	2
Parvoviridae							1/4		
Red-crowned crane parvovirus	4/6	2E-13–1E-10	28–41				1/4	3,00E-11	21
Canine bocavirus 3	1/6	7,00E-32	13						
Bat parvovirus	1/6	1,00E-22	10						
Bat bocavirus	1/6	3,00E-55	8						
Camel bocavirus 3	1/6	1,00E-35	7						
Murine bocavirus	1/6	8,00E-21	8						
Peribunyaviridae	*2/6*						1/4		
Orthobunyavirus sp.	2/6	1E-23–1E-22	2				1/4	1,00E-22	2
Picornaviridae	*1/6*						1/4		
Human Rhinovirus A	1/6	9,00E-51	2						
Porcine kobuvirus							1/4	5,00E-78	6
Sheep kobuvirus							1/4	2,00E-19	2

Poxviridae			
Orf virus	-	1E-50-6E-11	2-4

Supplementary Table S2. Viral blastn hits from rectal swabs collected from semi-domesticated Eurasian tundra reindeer herds in Sweden during late autumn 2017. Reindeer were sampled in herds from three regions (A, B, C).

Virus	Region A 08-12-2017			Region B 08-12-2017			Region C 16-11-2017		
	**Pools**	**e-value (min)**	**Read count**	**Pools**	**e-value (min)**	**Read count**	**Pools**	**e-value (min)**	**Read count**
Arenaviridae							3/3		
Lassa mammarenavirus				3/4	4E-48–2E-13	90–193	3/3	1E-78–4E-76	24–163
Guanarito mammarenavirus virus				1/4	9,00E-30	2	3/3	4E-51–2E-46	42–686
Flaviviridae	*1/2*						1/3		
Iguape virus	1/2	1,00E-41	2	2/4	3,00E-17	9	1/3	1,00E-31	9
Dengue virus				1/4	1E–28–8E-65	2–6			
Herpesviridae				4/4					
Alphaherpesviridae				4/4					
Various alphaherpesviruses				4/4	5E-27–6E-9	2-6			
Betaherpesvirinae				2/4					
Various betaherpesviruses				2/4	2E-19–2E-14	2-3			
Gammaherpesviridae				1/4					
Various gammaherpesviruses				1/4	1,00E-14	5			
Papillomaviridae				1/4					
Bovine papillomavirus				1/4	2,00E-30	6			
Pudu puda papillomavirus				1/4	6,00E-18	2			
Parvoviridae				X					
Red-crowned crane parvovirus				3/4	2E-12–2E-14	61–109			
Porcine bocavirus				1/4	9,00E-08	2			
Peribunyaviridae				1/4					
Orthobunyavirus sp.				1/4	1,00E-23	2	2/3	8E-25–1E-23	7–11
Ngari virus							1/3	1,00E-27	1
Simbu virus							1/3	8,00E-06	1
Shamonda virus							1/3	5,00E-08	1
Picobirnaviridae							2/3		
Marmot picobirnavirus							1/3	1,00E-08	2
Fox picobirnavirus							1/3	1,00E-06	2
Human picobirnavirus							1/3	1,00E-27	2
Macaque picobirnavirus							1/3	2,00E-10	2
Poxviridae				*1/4*					
Orf virus				1/4	1,00E-50	2			
Reoviridae	*1/2*								
Bovine rotavirus	1/2	1,00E-84	2	1/4	2,00E-31	8			
Other rotaviruses				2/4	3E-44–9E-8	2			
Small circular DNA viruses	*1/2*								

Circoviridae							1/3		
Bat assoc. cyclovirus							1/3	4,00E-27	2
Chimpanzee faeces assoc. c virus 1				2/4	2E-16–3E-16	7			
Porcine serum-assoc. circular virus				1/4	1,00E-15	6			
Other circoviruses				1/4	3,00E-10	4			
Genomoviridae							1/3		
Cattle blood-assoc. Gemycircular-virus							1/3	7,00E-12	1
Giant panda assoc. Gemycircular-virus				1/4	4,00E-29	3	1/3	1,00E-06	2
Other Genomoviruses				2/4	4E-60–2E-13	1-2			
Smacoviridae	1/2								
Goat assoc. porprismacovirus	1/2	6,00E-37	48						
Sheep faeces assoc. smacovirus 1	1/2	3,00E+23	48						
Porcine assoc. smacovirus	1/2	1,00E-07	15						
Odonata-assoc. circular virus-21	1/2	3,00E-09	4						
Alces alces faeces assoc. smacovirus	1/2	3,00E+16	2						
Sewage-assoc. circular DNA virus-9				1/4	2,00E-37	13			
Porcine assoc. porprismacovirus				2/4	7E-35–3E-23	4–10			
Turkey assoc. porprismacovirus 1				1/4	3,00E-13	5			
Macaca mulatta feces assoc. virus 2				2/4	1E-41–8E-29	2–4			
Other smacovirus				3/4	1E-31–	2–5			
Unclassified cressdnaviricota				1/4					
CRESS virus sp.				1/4	2,00E-52	19			
Unclassified ssDNA viruses				X					
Fly assoc. circular virus 6				1/4	1,00E-53	10			
Fly assoc. circular virus 7				1/4	7,00E-10	10			
Goat blood assoc. virus				1/4	9,00E-13	3			
Chicken stool-assoc. circular virus				2/4	5E-25–1E-13	2–3			
Other unclassified ssDNA virus				2/4	2E-7–8E-6	2			
Unclassified viruses	*1/2*						2/3		
Phitovirus	1/2	4,00E-10	2						
Statovirus							2/3	2E-17–2E-38	2–10

Supplementary Table S3. Viral blastn hits from nasal swabs collected from semi-domesticated Eurasian tundra reindeer herds in Sweden during late autumn 2016. Reindeer were sampled in herds from three regions (A, B, C).

Virus	Region A 08-11-2016			Region B 06-12-2016			Region C 01-11-2016		
	Pools	e-value (min)	Read count	Pools	e-value (min)	Read count	Pools	e-value (min)	Read count
Adenoviridae				1/4					
Bovine adenovirus type 3				1/4	3,00E-35	4			
Deer mastadenovirus B				1/4	3,00E-42	2			
Ovine adenovirus 6				1/4	8,00E-45	2			

Arenaviridae	*7/7*			4/4			8/8		
Guanarito mammarenavirus	7/7	3E-51–2E-48	84–518	4/4	2E-51–1E-48	165–429	8/8	1E-51–9E-43	10–276
Lassa mammarenavirus	7/7	1E-75–2E-52	7–26	4/4	2E-79–4E-70	15–28	7/8	3E-77–1E-54	7–26
Luna mammarenavirus	1/7	3,00E-29	1						
Flaviviridae	*2/7*			1/4			1/8		
Bovine viral diarrhea virus-1	2/7	1E-69–2E-43	2	1/4	6,00E-59	2	1/8	5,00E-53	2
Dengue virus				1/4	2,00E-06	2			
Herpesviridae							3/8		
Alphaherpesviridae							3/8		
Various alphaherpesviruses							3/8	4E-20–1E-6	2-3
Betaherpesvirinae							1/8		
Various betaherpesviruses							1/8	1,00E-09	3
Gammaherpesvirinae									
Bovine herpesvirus 6							2/8	7E-43–3E-30	1–57
Ovine/caprine herpesvirus 2							2/8	8E-37–4E-28	20–61
Alcelaphine herpesvirus 2							2/8	9E-43–1E-40	11–52
Alcelaphine herpesvirus 1							2/8	2E-44–1E-40	11–42
Other gammaherpesviruses							2/8	5E-27–6E-20	6–38
Paramyxoviridae	*1/7*						1/8		
Human Respirovirus 1	1/7	2,00E-48	2				1/8	2,00E-48	2
Parvoviridae	*2/7*			1/4			2/8		
Red-crowned crane parvovirus	2/7	5E-14–3E-10	2–26	1/4	2,00E-12	13	2/8	3E-17–3E-14	42–48
Peribunyaviridae	*2/7*			3/4			4/8		
Orthobunyavirus sp.	2/7	9E-24–4E-21	2-4	3/4	1E-22–6E20	2	4/8	1E-22–8E-25	2-6
Poxviridae	*2/7*			*1/4*					
Orf virus	2/7	2E-37–2E-26	2	1/4	6,00E-54	2	1/8	1,00E-41	3
White-tailed deer poxvirus							1/8	2,00E-35	2
Small circular DNA viruses									
Circoviridae							1/8		
Chimpanzee faeces assoc. c virus 1							1/8	6,00E-09	2
Genomoviridae	1/7								
Blackbird faeces-assoc. Gemycircular-virus	*1/7*	1,00E-07	2						
Unclassified ssDNA viruses	*1/7*								
Porcine serum-assoc. circular virus	*1/7*	7,00E-46	2						

Supplementary Table S4. Viral blastn hits from nasal swabs collected from semi-domesticated Eurasian tundra reindeer herds in Sweden during late autumn 2017. Reindeer were sampled in herds from three regions (A, B, C).

Virus	Region A 08-12-2017			Region B 08-12-2017			Region C 16-11-2017		
	**Pools**	**e-value (min)**	**Read count**	**Pools**	**e-value (min)**	**Read count**	**Pools**	**e-value (min)**	**Read count**
Arenaviridae				3/3			4/4		
Guanarito mammarenavirus virus	2/4	3E-39–2E-38	5–6	2/3	8E-48–1E-46	7-9	4/4	6E-49–7E-48	15–152

Lassa mammarenavirus	2/4	2E-69–1E-43	12–18	3/3	1E-70–2E-32	12–40	4/4	6E-77–3E-73	63–242
Flaviviridae							1/4		
Bovine viral diarrhea virus 1							1/4	1,00E-34	1
Dengue virus				1/3	2,00E-14	9			
Iguape virus				1/3	1,00E-35	2	1/4	5,00E-32	5
Herpesviridae	*2/4*						1/4		
Alphaherpesviridae				*1/3*					
Various alphaherpesviruses				1/3	4,00E-41	4			
Gammaherpesvirinae	*2/4*			2/3			1/4		
Bovine herpesvirus 6	2/4	4,00E-09	3					2,00E-13	4
Ovine/caprine herpesvirus 2								8,00E-24	3
Alcelaphine herpesvirus 1	2/4	2,00E-08	2					2,00E-29	2
Alcelaphine herpesvirus 2	2/4	2,00E-12	1					1,00E-22	4
Saimiriine herpesvirus 2				2/3	1E-15–2E-15	2			
Other gammagherpesviruses	2/4	1E-9–3E-9	1–2					6E-32–1E-8	1-2
Paramyxoviridae							1/4		
Human Respirovirus 1							1/4	1,00E-22	2
Parvoviridae	*2/4*			1/4			1/4		
Bovine parvovirus 3							1/4	3,00E-12	2
Red-crowned crane parvovirus	2/4	7E-13–1E-9	4-5	1/4	2,00E-12	13			
Ungulate bocaparvovirus 6							1/4	1,00E-06	1
Peribunyaviridae				1/3			4/4		
Orthobunyavirus sp.				1/3	1,00E-23	4	4/4	1E-25–2E-19	4-23
Picobirnaviridae							1/4		
Marmot picobirnavirus							1/4	2,00E-08	2
Poxviridae	*1/4*						1/4		
Orf virus	1/4	6,00E-20	2	1/3	4,00E-66	4	1/4	2,00E-49	2
Yoka poxvirus				1/3	1,00E-07	2			
Eptesipox virus				1/3	5,00E-06	2			
Reoviridae							1/4		
Kammavanpettai virus							1/4	2,00E-18	2
Small circular DNA viruses									
Circoviridae				1/3			1/4		
Bat assoc. circovirus 7							1/4	1,00E-20	1
Cattle blood-assoc. Gemycirculavirus 4				1/3	2,00E-06	1			
Porcine circovirus 3							1/4	8,00E-22	3
Genomoviridae							1/4		
Faeces assoc. Gemycircularvirus 21							1/4	2,00E-16	2
Giant panda assoc. gemycircularvirus				1/3	1,00E-85	13			
Pteropus assoc. gemycircularvirus 4				1/3	2,00E-12	1			
Tick gemycircularvirus							1/4	1,00E-17	2
Unclassified viruses							1/4		

Virus	Pools	e-value (min)	Read count	Pools	e-value (min)	Read count	Pools	e-value (min)	Read count
Statovirus D1							1/4	9,00E-12	2

Supplementary Table S5. Viral blastn hits from rectal swabs collected from semi-domesticated Eurasian tundra reindeer herds in Norway during Nov-Jan 2016-17. Reindeer were sampled in herds from three regions (A, B, C).

Virus	Region A 23-11-2016			Region B 26-01-2017			Region C 24-01-2017		
	**Pools**	**e-value (min)**	**Read count**	**Pools**	**e-value (min)**	**Read count**	**Pools**	**e-value (min)**	**Read count**
Arenaviridae	*3/3*						4/4		
Guanarito mammarenavirus	3/3	2E-51–4E-43	9-133	3/4	7E-44–2E-40	5–15	4/4	4E-53–2E-44	13–146
Lassa mammarenavirus	3/3	3E-72–1E-47	4–44	1/4	9,00E-71	8	4/4	4E-77–3E-72	7–47
Astroviridae				3/4					
Bovine astrovirus	1/3	7,00E-43	2	2/4	7E-95–4E-73	2–44			
Yak astrovirus				2/4	1E-104–3E-36	1–17			
Deer astrovirus	1/3	3,00E-16	2	2/4	1E-59–3E-53	15			
Sichuan takin astrovirus				2/4	4E-84–1E-66	3–11			
Other astroviruses				1/4	4,00E-53	3			
Caliciviridae									
Bovine calicivirus				2/4	7E-56–1E-7	2–38			
Other caliciviruses				2/4	4E-9–2E-6	1–5			
Flaviviridae									
Bovine viral diarrhea virus 1							1/4	7,00E-87	3
Dengue virus							1/4	2,00E-07	4
Herpesviridae	3/3			4/4			2/4		
Alphaherpesvirinae	3/3			4/4			2/4		
Various alphaherpesviruses	3/3	1E-21–3E-10	4	4/4	2E-33–5E-8	2–6	2/4	5E-31–6E-6	3–4
Betaherpesvirinae				1/4					
Various betaherpesviruses				1/4	3,00E-09	2			
Gammaherpesvirinae	2/3			2/4					
Various gammaherpesviruses	2/3	9E-29–2E-12	3–8	2/4	3E-23–5E-21	3–4			
Papillomaviridae				1/4			1/4		
Bos taurus papillomavirus 16				1/4	1,00E-21	2			
Bovine papillomavirus	1/3	1,00E-66	25				1/4	5,00E-59	16
Human papillomavirus	1/3	6,00E-13	9						
Pudu puda papillomavirus							1/4	2,00E-75	10
Ovine papillomavirus	1/3	4,00E-09	1				1/4	6,00E-20	3
Peribunyaviridae	*1/3*						X		
Orthobunyavirus sp.	1/3	1,00E-22	4				1/4	1,00E-23	3
Ngari virus							1/4	1,00E-19	1
Picobirnaviridae	*1/3*								
Marmot picobirnavirus	1/3	2,00E-27	2	2/4	2E-76–2E-50	2			
Other picobirnaviruses				2/4	2E-14–2E-6	2			
Picornaviridae							1/4		
Bovine Aichivirus B				1/4	1,00E-15	1			

Caprine kobuvirus				1/4	1,00E-115	44			
Cattle kobuvirus				2/4	2E-83–6E-64	4–27			
Other kobuviruses							1/4	2,00E-71	3
Porcine Aichivirus C				1/4	3,00E-30	2			
Porcine kobuvirus				2/4	4E-85–1E-52	2-53	1/4	4,00E-72	4
Poxviridae									
Cow poxvirus	1/3	1,00E-09	1						
Orf virus	1/3	2,00E-75	2				3/4	1E-105–2E-21	1–4
Orthopoxvirus Abatino				1/4	3,00E-09	3			
Other poxviruses				2/4	6E-25–7E-6	2–4	-	4E-14–2E-11	2
Squirrel poxvirus				3/4	1E-19–3E-11	1–4			
Reoviridae									
Human rotavirus A	1/3	1,00E-101	3	2/4	1E-134–1E-114	1–12			
Bovine rotavirus A				1/4	1,00E-138	4			
Feline rotavirus A	1/3	1,00E-59	1						
Porcine rotavirus A				2/4	1E-105–6E-45	2–4			
Other rotavirus A				3/4	1E-133–1E-78	1–4			
Small circular DNA viruses				4/4					
Circoviridae				1/4					
Chimpanzee faeces assoc. cc circovirus				1/4	3,00E-09	15			
Dromedary stool-assoc. circular virus				1/4	5,00E-14	5			
Other circoviruses				1/4	5,00E-20	3			
Genomoviridae				1/4					
Sewage derived gemycircularvirus 1				1/4	1,00E-18	4			
Smacoviridae				4/4			1/4		
Porcine feces assoc. smacoviruses				4/4	3E-55–5E-14	3–169			
Chimpanzee feces assoc. smacovirus				1/4	5,00E-07	54			
Alces alces faeces assoc. Smacovirus				2/4	5E-52–8E-50	8–27	1/4	3,00E-17	2
Sheep feces assoc. Smacoviruses				3/4	2E-51–3E-17	6–23			
Cattle blood-assoc. smacovirus				1/4	5,00E-33	29			
Macaque feces assoc. smacoviruses				3/4	4E-34–8E-13	7–19			
Other Smacoviruses				4/4	1E-39–3E-23	3–34			
Unclassified viruses	*1/3*								
Porcine serum-assoc. circular virus				3/4	2E-15–4E-15	3–96			
Chicken stool-assoc. circular virus				1/4	1,00E-22	33			
Rodent stool-assoc. Circular virus	1/3	1,00E-33	2						
Unidentified circular ssDNA virus				3/4	1E-47–3E-09	3–11			
Other unclassified viruses				1/4	1,00E-13	6			
Unclassified viruses							1/4		
Hubei partiti-like virus 55				1/4	6,00E-06	2			
Statovirus D1				1/4	1,00E-09	2	1/4	2,00E-13	2

Supplementary Table S6. Viral blastn hits from rectal swabs collected from semi-domesticated Eurasian tundra reindeer herds in Norway during Nov-Apr 2017-18. Reindeer were sampled in herds from three regions (A, B, C).

Viruses	Region A 23-11-2017			Region B			Region C 24-01-2018		
	**Pools**	**e-value (min)**	**Read count**	**Pools**	**e-value (min)**	**Read count**	**Pools**	**e-value (min)**	**Read count**
Alloherpesviridae	*1/4*								
Cyprinid herpesvirus 3	1/4	3,00E-49	8						
Arenaviridae	*1/4*						1/4		
Lassa mammarenavirus	1/4	7,00E-07	2				1/4	2,00E-06	2
Flaviviridae				1/3					
Bovine viral diarrhea virus 1									
Iguape virus				1/3	1,00E-41	2			
Herpesviridae	*4/4*								
Alphaherpesvirinae							*1/4*		
Macacine alphaherpesvirus 1							1/4	2,00E-09	2
Betaherpesvirinae							*1/4*		
Various betaherpesviruses							1/4	1,00E-21	2
Gammaherpesvirinae	*4/4*								
Alcelaphine herpesvirus 1	1/4	5,00E-21	2						
Alcelaphine herpesvirus 2	1/4	4,00E-24	4						
Bovine herpesvirus 6	2/4	7E-29–1E-13	2-3				1/4	2,00E-10	2
Ovine herpesvirus 2	1/4	5,00E-07	2				1/4	2,00E-24	2
Other gammaherpesviruses	3/4	9E-29–5E-6	2-10				1/4	1,00E-16	4
Papillomaviridae							2/4		
Bovine papillomaviruses							1/4	5,00E-59	182
Human papilloma virus 5							1/4	5,00E-10	10
Puda Puda papillomavirus 1							1/4	6,00E-12	4
Other papillomaviruses							1/4	4,00E-16	6
Parvoviridae							2/4		
Red-crowned crane parvovirus							2/4	1E-15–4E-15	17–18
Tasmanian devil-assoc. Chapparvovirus				1/3	8,00E-16	3			
Other parvoviruses				2/3	4E-12–5E-10	2–3			
Picobirnaviridae				1/3			4/4		
Human picobirnavirus	3/4	1E-72–9E-24	2–11	1/3	3,00E-44	2	1/4	1,00E-86	6
Dromedary picobirnavirus	1/4	2,00E-25	4				2/4	2E-74–3E-22	2–6
Equine Picobirnavirus	1/4	4,00E-06	2						
Macaque picobirnavirus							4/4	8E-36–1E-18	2–10
Marmot picobirnavirus							3/4	6E-90–7E-15	2–6
Gorilla picobirnavirus							2/4	2E-6–9E-6	3–4
Genet fecal picobirnavirus							1/4	1,00E-91	14
Other picobirnaviruses							2/4	3e-28–2E-10	2
Picornavirales (Unclassified)	2/4						2/4		
Posavirus 1	2/4	1E-8–2E-8	2–3				2/4	5E-8–2E-8	7–10

Picornaviridae	*1/4*								
Caprine kobuvirus							1/4	1,00E-59	10
Porcine kobuvirus	1/4	1,00E-134	6				3/4	1E-117–3E-27	2–14
Pneumoviridae							2/4		
Swine pneumovirus 57							2/4	2E-6–2E-8	2
Poxviridae							1/4		
Canarypoxvirus							1/4	3,00E-06	4
Small circular DNA viruses				3/3			4/4		
Circoviridae				3/3			2/4		
Chimpanzee faeces assoc. virus 1	1/4	4,00E-08	2						
CRESS virus sp.	3/4	1E-17–2E-8	2	3/3	1E-117–1E-55	2–4228			
Various circoviruses							2/4	2E-20–2E-7	2–8
Genomoviridae									
Dragonfly-assoc. circular virus 2							1/4	1,00E-35	11
Giant panda assoc. gemycircularvirus	1/4	2,00E-20	6						
Gopherus assoc. genomovirus 1				1/3	1,00E-11	13			
Insect assoc. genomoviruses	1/4	2,00E-52	24	1/3	2,00E-89	66			
Lynx faeces assoc. genomovirus							1/4	1,00E-16	1
Momordica charantia assoc. virus							1/4	2,00E-12	4
NZ fur seal faeces assoc. virus				1/3	8,00E-24	19			
Pteropus assoc. gemycircularvirus 4	1/4	3,00E-11	6						
Sewage-assoc. virus							1/4	1,00E-12	8
Snowshoe hare faeces assoc. genomovirus	1/4	8,00E-12	8	1/3	3,00E-07	44			
Other gemycircularviruses	1/4	2,00E-24	4	2/3	2E-23–3E-15	2–10			
Other genomoviruses				1/3	4,00E-09	3			
Smacoviridae	*2/4*			3/3			4/4		
Alces alces faeces assoc. smacovirus	2/4	4,00E-06	4						
Cattle-blood assoc. smacovirus							1/4	8,00E-08	10
Chimpanzee feces assoc. smacovirus							4/4	6E-13–3E-6	10–20
Goat assoc. porprismacovirus	2/4	8,00E-12	5						
Insect assoc. smacoviruses							1/4	2,00E-27	14
Odonata-assoc. circular virus-5	2/4	1,00E-18	3						
Porcine assoc. smacovirus				3/3	1E-118–5E-50	1–1838			
Porcine stool assoc. smacovirus							2/4	9E-27–9E-14	12–15
Sheep faeces assoc. smacovirus 1	2/4	4,00E-18	15	2/3	3E-16–1E-8	2–6			
Sheep/goat assoc. smacovirus							3/4	3E-24–2E-11	2–4
Other smacoviruses				3/3	3E-62–9E-17	6–9	1/4	2,00E-30	15
Unclassified ssDNA viruses	*2/4*			1/3					
Unidentified circular ssDNA viruses							1/4	2,00E-08	19
Bovine faeces assoc. DNA virus 1							1/4	-	-
Various unclassified ssDNA viruses	2/4	1E-26–2E-23	6-14	1/3	1,00E-36	36			
Unclassified environmental viruses				1/3			1/4		

Uncultured viruses	1/3	1,00E-29	4	1/4	3,00E-14	8
Unclassified RNA viruses	1/3					
Beihai narna-like virus 7				1/4	3,00E-09	4
Hubei narna-like virus 11	1/3	2,00E-06	2			
Statovirus D1				3/4	2E-50–9E-7	5–26

Supplementary Table S7. Viral blastn hits from nasal swabs collected from semi-domesticated Eurasian tundra reindeer herds in Norway during Nov-Jan 2016-17. Reindeer were sampled in herds from three regions (A, B, C).

**Virus**	**Region A 23-11-2016**			**Region B 26-01-2017**			**Region C 24-01-2017**		
	**Pools**	**e-value (min)**	**Read count**	**Pools**	**e-value (min)**	**Read count**	**Pools**	**e-value (min)**	**Read count**
Adenoviridae							1/5		
California sea lion adenovirus 1							1/5	8,00E-06	2
Arenaviridae	*7/7*			6/6					
Guanarito mammarenavirus	1/7	2,00E-44	27	1/6	1,00E-40	9	2/5	4E-43–3E-46	7–11
Lassa mammarenavirus	7/7	3E-79–3E-54	2–30	6/6	1E-76–1E-46	6–21	4/5	2E-76–5E-53	7–34
Astroviridae				2/6					
Hainan astro-like virus 2				2/6	9E-8–1E-7	2–4			
Flaviviridae				4/6					
Bovine viral diarrhea virus 1	*2/7*	1E-123–2E-55	2	4/6	2E-45–3E-29	1–3	2/5	1E-32–9E-24	1
Dengue virus	1/7	2,00E-19	3				1/5	5,00E-14	11
West Nile virus	1/7	7,00E-06	1						
Herpesviridae									
Alphaherpesvirinae	*1/7*			2/6			2/5		
Various alphaherpesviruses	1/7	5,00E-07	3	2/6	1E-8–6E-6	2–3	2/5	3E-23–4E-21	2
Betaherpesvirinae	*2/7*			1/6			3/5		
Cervid herpesvirus 3				1/6	1,00E-52	2			
Various betaherpesviruses	2/7	2E-31–9E-24	7–22				3/5	2E-39–6E-14	4–5
Papillomaviridae	*1/7*			1/6					
Betapapillomavirus 1				1/6	1,00E-135	2			
Reindeer papillomavirus	1/7	1,00E-129	2						
Paramyxoviridae	*1/7*			1/6					
Human respirovirus 1	1/7	3,00E-23	2	1/6	2,00E-13	2			
Human respirovirus 3							1/5	1,00E-60	15
Bovine respirovirus 3							2/5	3,00E-62	9
Caprine respirovirus 3							2/5	2,00E-24	2
Peribunyaviridae	*2/7*			2/6			1/5		
Orthobunyavirus sp.	2/7	1E-22–5E-20	2–3	2/6	1E-22–5E-21	4	1/5	1,00E-24	2
Poxviridae	*2/7*			1/6			1/5		
Orf virus	2/7	2E-81–9E-49	2–4	1/6	1,00E-59	2			
Various poxviruses							1/5	1,00E-10	2
Reoviridae				1/6					
Rotavirus A				1/6	2,00E-60	2			

Small circular DNA viruses	1/7								
Genomoviridae	*1/7*								
Sewage derived gemycircularvirus	1/7	1,00E-16	2						
Smacoviridae									
Camel assoc. Smacovirus				1/6	2,00E-38	2			
Sheep faeces assoc. smacovirus 1				1/6	1,00E-27	2			

Supplementary Table S8. Viral blastn hits from nasal swabs collected from semi-domesticated Eurasian tundra reindeer herds in Norway during Nov-Apr 2017-18. Reindeer were sampled in herds from three regions (A, B, C).

Virus	Region A 23-11-2017			Region B			Region C 24-01-2018		
	Pools	e-value (min)	Read count	Pools	e-value (min)	Read count	Pools	e-value (min)	Read count
Arenaviridae							1/4		
Lassa mammarenavirus				2/4	2E-7–2E-6	2–4	1/4	5,00E-08	2
Luna mammarenavirus				1/4	2,00E-39	1			
Flaviviridae				3/4					
Bovine viral diarrhea virus 1							1/4	8,00E-06	1
Dengue virus							1/4	1,00E-08	2
Iguape virus				3/4	1E-69–6E-60	8–47	3/4	3E-63–2E-39	2–8
Herpesviridae				1/4			4/4		
Alphaherpesvirinae							1/4		
Various alphaherpesviruses							1/4	8,00E-44	3
Betaherpesvirinae							4/4		
Human herpesvirus 6							4/4	6E-39–2E-15	2–92
Human herpesvirus 7							2/4	1E-47–9E-41	33–83
Macaca nemestrina herpesvirus 7							2/4	1E-29–2E-25	51–276
Various betaherpesviruses							3/4	9E-65–2E-15	8–124
Gammaherpesvirinae				1/4			4/4		
Ovine herpesvirus 2	1/3	4,00E-35	2				4/4	4E-66–5E-8	1–54453
Alcelaphine herpesvirus 2	2/3	3E-42–5E-20	2	1/4	4,00E-28	2	3/4	6E-71–6E-30	1–36279
Alcelaphine herpesvirus 1							2/4	7E-64–3E-8	2–36702
Bovine herpesvirus 6							2/4	2E-51–1E-12	3–33644
Other gammaherpesviruses							3/4	1E-98–2E-9	1–25354
Unclassified herpesviruses									
Ovine herpesvirus 1							1/4	7,00E-32	1217
Rhinolophus ferrumequinum herpesvirus 1							2/4	2E-13–5E-12	16–48
Hipposideros diadema herpesvirus							1/4	1,00E-21	32
Other unclassified herpesviruses							1/4	5,00E-07	15
Papillomaviridae				4/4			3/4		
Bovine papillomaviruses				2/4	3E-49–4E-13	2–42	3/4	3E-47–6E-26	4–89
Human papillomaviruses				3/4	9E-18–9E-7	2–24	1/4	7,00E-25	41
Cervus elaphus papillomaviruses				2/4	3E-8–4E-8	2–5			

Other papillomaviruses				1/4	9E-12–5E-25	2–7	1/4	5,00E-08	19
Parvoviridae	*1/3*						4/4		
Red-crowned crane parvovirus	1/3	5,00E-15	8				4/4	2E-16–3E-9	9–28
Picobirnaviridae	1/3								
Human picobirnavirus	1/3	4,00E-50	4	1/4	2,00E-61	4			
Lysoka partiti-like virus				1/4	6,00E-07	4			
Picornavirales (Unclassified)	*1/3*			1/4					
Posavirus 1	1/3	3,00E-11	2	1/4	1,00E-07	3			
Polyomaviridae							1/4		
Human polyomavirus 12							1/4	4,00E-06	6
Miniopterus schreibersii polyomavirus 1							1/4	3,00E-06	5
Dolphin polyomavirus 1							1/4	6,00E-14	4
Other Polyomaviruses							1/4	6,00E-14	13
Reoviridae									
Bluetongue virus				1/4	2,00E-07	2			
Porcine rotavirus				1/4	2,00E-28	2			
Small circular DNA viruses									
Genomoviridae									
Lynx canadensis faeces assoc. virus				1/4	3,00E-09	2			
Pteropus assoc. gemycircularvirus 3	1/3	1,00E-12	2						
Sewage assoc. Gemycircularvirus 7b	1/3	7,00E-06	2						
Spider assoc. circular virus 1				1/4	2,00E-67	10			
Circoviridae									
Porcine circovirus 3				2/4	1E-29–4E-29	2–11			
Other circoviruses				1/4	4,00E-10	6			
CRESS virus sp.				2/4	4E-10–6E-9	2–3			
Smacoviridae									
Lemur assoc. porprismacovirus 1				1/4	3,00E-26	3			
Macaque feces assoc. virus 2				1/4	3,00E-07	2			
Other smacoviruses				1/4	2,00E-14	4			
Unidentified ssDNA viruses				1/4					
Unidentified circular ssDNA virus				1/4	7,00E-57	4			
Unclassified environmental viruses	*2/3*								
Uncultured viruses	2/3	3E-44–3E-60	4						
Unclassified RNA viruses				1/4					
Hainan astro-like virus 2				1/4	7,00E-08	40			
Hubei diptera virus 20				1/4	2,00E-07	2			

Supplementary Table S9. Viral blastn hits from rectal swabs collected from semi-domesticated Eurasian tundra reindeer herds in Finland during Dec-Feb 2016-17. Reindeer were sampled in herds from three regions (A, B, C).

Virus	Region A 14-12-2016			Region B 30-01-2017			Region C 02-02-2017		
	Pools	e-value (min)	Read count	Pools	e-value (min)	Read count	Pools	e-value (min)	Read count

Alloherpesviridae				1/4					
Anguillid herpesvirus				1/4	9,00E-23	2			
Arenaviridae	*1/4*			4/4			1/4		
Lassa mammarenavirus	1/4	1,00E-08	4	4/4	2E-13–1E-9	3-14	1/4	3E-11–1E-8	5–16
Astroviridae				1/4			1/4		
Bovine astrovirus				1/4	1,00E-46	8	1/4	2,00E-17	2
Porcine astrovirus 2				1/4	9,00E-20	6			
Other astroviruses				1/4	6,00E-16	4			
Flaviviridae	4/4			4/4			3/4		
Iguape virus	4/4	3E-70–5E-61	8–42	4/4	7E-73–1E-41	2–32	3/4	4E-69–4E-62	3–42
Parvoviridae	*4/4*			4/4			4/4		
Red-crowned crane parvovirus	4/4	9E-10–7E-7	2–3	4/4	1E-14–3E-11	7–9	4/4	2E-13–5E-8	2–8
Cutavirus				1/4	2,00E-09	2			
Picobirnaviridae							1/4		
Dromedary picobirnavirus				1/4	3,00E-07	2			
Lysoka partiti-like virus				1/4	2,00E-10	4	1/4	4,00E-30	2
Marmot picobirnavirus							1/4	9,00E-10	2
Picobirnavirus sp.							1/4	2,00E-14	2
Picornaviridae				1/4					
Rodent hepatovirus A				1/4	2,00E-51	14			
Human hepatovirus A				1/4	8,00E-19	9			
Woodchuck hepatovirus A				1/4	2,00E-35	6			
Other hepatoviruses A				1/4	1,00E-33	4			
Poxviridae	*1/4*								
Orf virus	1/4	3,00E-44	2						
Small circular DNA viruses									
Smacoviridae							1/4		
Cattle blood-assoc. circular virus				1/4	1,00E-54	27	1/4	7,00E-13	19
Chimpanzee assoc. porprismacovirus							1/4	4,00E-06	3
Camel assoc. porprismacovirus				2/4	8E-88–3E-78	4–7			
Other smacoviruses				1/4	1,00E-72	9			
Genomoviridae				1/4					
Cattle plasma-assoc. gemycircular-virus				1/4	3,00E-45	9			
Gila monster-assoc. gemycircularvirus				1/4	6,00E-66	3			
Unclassified ssDNA viruses									
Unidentified circular ssDNA virus							-	7,00E-12	6
Unclassified environmental viruses				1/4					
Uncultured viruses				1/4	5,00E-12	2			
Unclassified viruses				1/4					
Pecovirus				1/4	1,00E-08	2			

Supplementary Table S10. Viral blastn hits from rectal swabs collected from semi-domesticated Eurasian tundra reindeer herds in Finland during Oct-Nov 2017. Reindeer were sampled in herds from three regions (A, B, C).

Virus	Region A 18-11-2017			Region B 26-10-2017			Region C 30-10-2017		
	**Pools**	**e-value (min)**	**Read count**	**Pools**	**e-value (min)**	**Read count**	**Pools**	**e-value (min)**	**Read count**
Adenoviridae							1/4		
Bovine adenoviruses							1/4	3,00E-48	129
Simian adenoviruses							1/4	1,00E-23	88
Porcine adenoviruses							1/4	7,00E-76	69
Bat adenoviruses							1/4	3,00E-31	30
Human adenoviruses							1/4	2,00E-27	21
Other adenoviruses							1/4	3,00E-49	29
Parvoviridae							1/4		
Red-crowned crane parvovirus							1/4	1,00E-14	2
Picobirnaviridae	3/4			2/4					
Human picobirnavirus (pseudogene)	3/4	3E-64–5E-15	2–13	2/4	9E-50–1E-42	2–3			
Small circular DNA viruses									
Genomoviridae				1/4					
Momordica charantia assoc. Gemycircular-virus							1/4	3,00E-81	1
Pteropus assoc. gemycircularvirus 3							1/4	2,00E-89	1
Tubeweb spider assoc. circular virus 1				1/4	5,00E-34	2			
Smacoviridae				1/4					
Sheep faeces assoc. Smacovirus 1				1/4	6,00E-95	2			
Unclassified environmental viruses							1/4		
Uncultured viruses							1/4	6,00E-29	2

Supplementary Table S11. Viral blastn hits from nasal swabs collected from semi-domesticated Eurasian tundra reindeer herds in Finland during Dec-Feb 2016-17. Reindeer were sampled in herds from three regions (A, B and C).

Virus	Region A 14-12-2016			Region B 30-01-2017			Region C 02-02-2017		
	**Pools**	**e-value (min)**	**Read count**	**Pools**	**e-value (min)**	**Read count**	**Pools**	**e-value (min)**	**Read count**
Adenoviridae	*1/4*								
Bovine adenovirus 3	1/4	2,00E-94	14						
Other adenoviruses	1/4	5,00E-65	4						
Alloherpesviridae	*4/4*						1/7		
Anguillid herpesvirus 1	4/4	5E-20–6E-14	3-5				1/7	1,00E-16	3
Arenaviridae									
Guanarito mammarenavirus							4/7	6E-52–2E-38	4–268
Lassa mammarenavirus							4/7	1E-78–5E-65	2–38
Flaviviridae	*1/4*								
Bovine viral diarrhea virus 1							2/7	3E-99–6E-33	2-3
Dengue virus	1/4	3,00E-17	3				1/7	1,00E-09	3

Iguape virus							4/7	1E-63–3E-35	2-11
Herpesviridae	*1/4*			1/4			3/7		
Alphaherpesvirinae							1/7		
Various alphaherpesviruses							1/7	9,00E-19	3
Betaherpesvirinae							1/7		
Panine herpesvirus 2							1/7	5,00E-08	4
Gammaherpesvirinae	*1/4*			1/4					
Ovine herpesvirus 2	1/4	2,00E-39	54				2/7	1E-53–1E-21	5–15
Human herpesvirus 4				1/4	6,00E-06	1			
Bovine herpesvirus 6	1/4	6,00E-39	51	1/4	2,00E-08	1	2/7	2E-38–2E-20	7–11
Alcelaphine herpesvirus 1	1/4	2,00E-46	43				2/7	8E-44–7E-38	7–9
Alcelaphine herpesvirus 2	1/4	3,00E-55	46				1/7	5,00E-59	5
Reindeer gammaherpesvirus							1/7	1,00E-98	2
Other gammaherpesviruses	1/4	1,00E-40	27				3/7	2E-20–6E-6	1–5
Paramyxoviridae							2/7		
Human respirovirus							2/7	6E-26–4E-16	2–6
Parvoviridae							1/7		
Mink bocavirus							1/7	5,00E-42	2
Pneumoviridae	1/4								
Swine pneumovirus 57	1/4	2,00E-07	2						
Poxviridae				1/4					
Orf virus				1/4	8,00E-51	2			

Supplementary Table S12. Viral blastn hits from nasal swabs collected from semi-domesticated Eurasian tundra reindeer herds in Finland during Oct-Nov 2017. Reindeer were sampled in herds from three regions (A, B, C).

Virus	Region A 18-11-2017			Region B 26-10-2017			Region C 30-10-2017		
	**Pools**	**e-value (min)**	**Read count**	**Pools**	**e-value (min)**	**Read count**	**Pools**	**e-value (min)**	**Read count**
Arenaviridae							1/4		
Guanarito mammarenavirus	1/4	1,00E-37	2				1/4	3,00E-17	2
Lassa mammarenavirus	1/4	1,00E-09	2						
Flaviviridae				1/4			1/4		
Bovine viral diarrhea virus 1				1/4	7,00E-38	3			
Dengue virus							1/4	3,00E-11	2
Iguape virus									
Herpesviridae									
Alphaherpesvirinae	*1/4*			2/4			1/4		
Cercopithecine herpesvirus 1	1/4	4,00E-06	2	1/4	4,00E-06	1			
Felid herpesvirus 1	1/4	7,00E-07	3	1/4	4,00E-09	1			
Human herpesvirus 3							1/4	5,00E-06	1
Suid herpesvirus 1							1/4	9,00E-23	1
Betaherpesvirinae							1/4		
Human herpesvirus 7							1/4	4,00E-22	1

Macaca nemestrina herpesvirus 7							1/4	1,00E-22	1
Gammaherpesvirinae	*1/4*			1/4					
Alcelaphine herpesvirus 1				1/4	3,00E-43	6			
Alcelaphine herpesvirus 2				1/4	3,00E-37	5			
Ovine herpesvirus 2				1/4	9,00E-28	4			
Saimiriine herpesvirus 2	1/4	2,00E-15	2						
Other gammaherpesviruses				1/4	8,00E-12	8			
Papillomaviridae									
Bovine papillomavirus							1/4	6,00E-09	2
Bovine papillomavirus 16							1/4	2,00E-50	1
Picobirnaviridae	1/4						2/4		
Human picobirnavirus (pseudogene)	1/4	7,00E-57	2				2/4	4E-53–1E-44	3–4

Supplementary Table S13. Viral blastn hits from rectal swabs collected from wild Eurasian tundra reindeer in Iceland in August 2017 and August 2018. Swab samples were collected during the hunting season and from all reindeer hunting districts.

**Virus**	**Region A Aug-17**			**Region A Aug-18**		
	**Pools**	**e-value (min)**	**Read count**	**Pools**	**e-value (min)**	**Read count**
Alloherpesviridae				1/4		
Cyprinid herpesvirus 3				1/4	1,00E-14	3
Arenaviridae	*2/3*			1/4		
Guanarito mammarenavirus	2/3	4E-39–4E-35	2–4			
Lassa mammarenavirus				1/4	5E-40–6E-13	5–32
Flaviviridae						
Bovine viral diarrhea virus 1	1/3	2,00E-19	1			
Dengue virus 1	1/3	2,00E-14	7			
Herpesviridae				2/4		
Alphaherpesvirinae						
Bovine herpesvirus type 1				1/4	6,00E-06	1
Equid herpesvirus 4				2/4	5E-8–7E-6	1
Human alphaherpesvirus 1				2/4	1E-20–1E-9	1–2
Macacine alphaherpesvirus 1				2/4	5E-27–2E-20	1–7
Suid herpesvirus 1				2/4	4E-15–7E-13	1
Gammaherpesvirinae						
Human herpesvirus 4				2/4	6E-20–4E-10	5–9
Human herpesvirus 8				1/4	6,00E-07	1
Macaca nemestrina rhadinovirus 2				1/4	7,00E-13	1
Parvoviridae				4/4		
Bocaparvovirus sp.				4/4	8E-14–1E-6	5–18
Red-crowned crane parvovirus				3/4	5E-15–9E-12	17–21
Peribunyaviridae						
Ngari virus				2/4	1,00E-26	2
Orthobunyavirus sp.				1/4	1,00E-09	2

Picobirnaviridae	1/4		
Chicken picobirnavirus	1/4	2,00E-08	2
Picornavirales (Unclassified)	1/4		
Posavirus 1	1/4	5,00E-08	2
Pneumoviridae	1/4		
Swine pneumovirus 57	1/4	2,00E-07	8
Poxviridae	1/4		
Akhmeta virus	1/4	5,00E-06	2
Small circular DNA viruses			
Circoviridae			
CRESS virus sp	2/4	2E-47–4E-22	33–253
Feline cyclovirus	1/4	1,00E-06	11
Genomoviridae			
Alces alces faeces assoc. genomovirus	2/4	3E-11–3E-6	2–8
Giant panda assoc. gemycircularvirus	1/4	1,00E-97	3
Insect-assoc. genomoviruses	1/4	3,00E-49	15
Lama faeces assoc. gemycircularvirus 21	2/4	2E-38–2E-26	4
Lupine feces-assoc. gemycircularvirus 1	1/4	6,00E-12	2
Lynx faeces assoc. genomovirus	1/4	5,00E-16	2
Sewage-assoc. gemycircularviruses	1/4	2,00E-58	17
Smacoviridae			
Bovine faeces assoc. smacoviruses	3/4	2E-50–1E-6	2–55
Odonata-assoc. smacoviruses	2/4	3E-21–5E-8	6–197
Porcine assoc. smacoviruses	3/4	8E-62–1E-35	2–378
Sheep/goat faeces assoc. smacoviruses	3/4	4E-28–5E-15	15–22
Other smacoviruses	3/4	2E-75–1E-16	2–64
Unclassified ssDNA viruses	1/4		
Bovine faeces assoc. circular DNA	1/4	6,00E-33	132
Cyanoramphus nest assoc. circular K DNA virus	1/4	4,00E-16	2
Rodent stool-assoc. circular genome virus	1/4	5,00E-07	71
Unidentified circular ssDNA virus	1/4	2,00E-16	174

Supplementary Table S14. Viral blastn hits from nasal swabs collected from wild Eurasian tundra reindeer in Iceland in August 2017 and August 2018. Swab samples were collected during the hunting season and from all reindeer hunting districts.

**Virus**	**Region A Aug-17**			**Region A Aug-18**		
	**Pools**	**e-value (min)**	**Read count**	**Pools**	**e-value (min)**	**Read count**
Arenaviridae	*8/8*			5/5		
Guanarito mammarenavirus	8/8	6E-75–7E-66	10–72			
Lassa mammarenavirus	8/8	1E-53–5E-46	36–235	5/5	5E-75–1E-10	2–31
Flaviviridae	*X*			*X*		
Bovine viral diarrhea virus 1	2/8	7E-38–2E-19	1			
Bovine viral diarrhea virus 2	1/8	3,00E-87	1			

Iguape virus	5/8	4E-54–3E-23	2–8	X	1E-63–3E-42	2–15
Herpesviridae	*X*					
Alphaherpesvirinae	*X*					
Macacine alphaherpesvirus 1	1/8	2,00E-08	2			
Suid herpesvirus 1	3/8	7E-38–4E-14	2			
Betaherpesvirinae				1/5		
Elephant endotheliotropic herpesvirus 4				1/5	5,00E-08	1
Human herpesvirus 4				1/5	2,00E-06	1
Human betaherpesvirus 6A				1/5	6,00E-07	1
Coronaviridae				1/5		
Python nidovirus				1/5	1,00E-40	4
Papillomaviridae	*X*					
Bovine papillomavirus (various types)	1/8	6,00E-58	12			
Human papillomavirus (various types)	2/8	1E-147–1E-17	2–6			
Rangifer papillomavirus	1/8	7,00E-19	1			
Paramyxoviridae	*1/8*					
Human respirovirus 1	1/8	1,00E-23	2			
Parvoviridae				5/5		
Red-crowned crane parvovirus				5/5	1E-14–8E-12	26–44
Peribunyaviridae	*3/8*			2/5		
Orthobunyavirus sp.	3/8	1E-22–6E-13	2–4	2/5	2,00E-22	2–4
Picobirnaviridae				1/5		
Lysoka partiti-like virus				1/5	4,00E-09	2
Picornavirales (Unclassified)				1/5		
Posavirus 1				1/5	6,00E-07	2
Poxviridae						
Goat poxvirus	1/8	8,00E-06	2			
Shearwaterpoxvirus	1/8	7,00E-33	4			
Small circular DNA viruses	1/8			1/5		
Circoviridae	*1/8*			1/5		
CRESS virus sp				1/5	1,00E-20	2
Feline cyclovirus	1/8	8,00E-06	2			

Supplementary Table S15. Viral blastn hits from rectal swabs collected from semi-domesticated Eurasian tundra reindeer herds in Yakutia, Russia, in December 2017 and November 2019. Reindeer were sampled in herds from three regions (A, B, C).

Virus	Region A 12-2017			Region B 11-2019			Region C 11-2019		
	Pools	e-value (min)	Read count	Pools	e-value (min)	Read count	Pools	e-value (min)	Read count
Arenaviridae	*1/1*								
Guanarito mammarenavirus	1/1	2,00E-50	55						
Lassa mammarenavirus	1/1	9,00E-43	11						
Luna mammarenavirus	1/1	1,00E-33	1						
Flaviviridae	*1/1*			*1/4*					

Iguape virus	1/1	9,00E-36	2	1/4	2,00E-65	12	1/4	2,00E-44	6
Dengue virus							1/4	3,00E-11	5
Herpesviridae									
Alphaherpesvirinae				1/4					
Macacine alphaherpesvirus				1/4	2,00E-06	2			
Betaherpesvirinae				1/4					
Human betaherpesvirus 6B				1/4	2,00E-11	2			
Parvoviridae	*1/1*						1/4		
Porcine bocavirus	1/1	1,00E-10	2						
Red-crowned crane parvovirus	1/1	6,00E-07	2				1/4	4,00E-10	4
Peribunyaviridae	*1/1*								
Orthobunyavirus sp.	1/1	1,00E-22	2						
Picobirnaviridae									
Dromedary picobirnavirus				3/4	8E-80–9E-46	2–4	2/4	6E-55–6E-27	2–5
Human picobirnavirus				1/4	8E-80–9E-46	2			
Macaque picobirnavirus				1/4	2,00E-47	2	2/4	9E-56–4E-47	1–5
Marmot picobirnavirus							2/4	1E-72–8E-63	1–3
Roe-deer picobirnavirus							2/4	2E-29–7E-20	2
Other picobirnavirus				1/4	1,00E-08	2	3/4	1E-23–2E-13	4
Picornavirales (Unclassified)				*2/4*					
Posavirus 1				2/4	2E-8–5E-8	2–12			
Picornaviridae	*1/1*			*2/4*					
Hedgehog hepatovirus	1/1	1,00E-11	1						
Hepatitis A virus	1/1	6,00E-77	10						
Porcine kobuvirus	1/1	2,00E-42	2	2/4	2E-64–3E-43	4			
Rodent hepatovirus	1/1	2,00E-58	2						
Poxviridae				1/4					
Cowpoxvirus				1/4	4,00E-06	2			
Fowlpoxvirus				1/4	1,00E-06	2			
Unclassified viruses							1/4		
Beihai narna-like virus 7							1/4	8,00E-11	23
Porcine feces assoc. IAS virus like virus				1/4	4,00E-21	2			
Statovirus D1				1/4	3,00E-17	13			
Wenzhou narna-like virus 4							1/4	1,00E-07	8

Supplementary Table S16. Viral blastn hits from nasal swabs collected from semi-domesticated Eurasian tundra reindeer herds in Russia in December 2017 and November 2019. Reindeer were sampled in herds from three regions (A, B, C).

**Virus**	**Region A 12-2017**			**Region B 11-2019**			**Region C 11-2019**		
	**Pools**	**e-value (min)**	**Read count**	**Pools**	**e-value (min)**	**Read count**	**Pools**	**e-value (min)**	**Read count**
Arenaviridae	*1/1*			2/4					
Guanarito mammarenavirus	1/1	1,00E-40	4						

Lassa mammarenavirus	1/1	3,00E-11	2	2/4	8E-23–5E-7	3–4			
Flaviviridae	*1/1*								
Classical swine fever virus	1/1	2,00E-31	2						
Dengue virus							1/4	6,00E-14	8
Iguape virus				-	4E-69–2E-66	4–11	3/4	5E-54–4E-13	2–33
Herpesviridae									
Alphaherpesvirinae				*1/4*					
Canine herpesvirus				1/4	9,00E-08	2			
Gammaherpesvirinae				*1/4*					
Alcelaphine herpesvirus 1				1/4	1,00E-29	13			
Alcelaphine herpesvirus 2				1/4	1,00E-19	5			
Bovine herpesvirus 6				1/4	4,00E-28	11			
Microtus agrestis rhadinovirus 1				1/4	8,00E-11	2			
Ovine herpesvirus 2				1/4	2,00E-11	4			
Papillomaviridae	*1/1*								
Bovine papillomavirus 20	1/1	8,00E-08	2						
Human papillomavirus (various types)	1/1	1,00E-143	6						
Paramyxoviridae							*1/4*		
Human respirovirus 1							1/4	8,00E-30	2
Parvoviridae	*1/1*								
Porcine bocavirus							1/4	9,00E-10	4
Red-crowned crane parvovirus	1/1	2,00E-08	2				1/4	2,00E-12	5
Peribunyaviridae							1/4		
Simbu orthobunyavirus							1/4	2,00E-09	2
Picornavirales (Unclassified)				*1/4*					
Posavirus 1				1/4	2,00E-08	4			
Picobirnaviridae	*1/1*								
Dromedary picobirnavirus				2/4	7E-49–5E-34	2			
Human picobirnavirus				2/4	8E-53–2E-7	1–9			
Human picobirnavirus pseudogene	1/1	3,00E-61	31						
Gorilla picobirnavirus				2/4	2E-55–6E-25	1–9			
Other picobirnaviruses				3/4	2E-55–2E-7	1–6			
Picornaviridae							*1/4*		
Bovine rhinitis A							1/4	8,00E-28	38
Bovine rhinitis B							1/4	2,00E-11	9
Foot-and-mouth disease virus type A							1/4	1,00E-10	6
Unclassified viruses				*X*					
Porcine feces assoc. IAS virus like virus				1/4	5,00E-13	2			
Statovirus D1				2/4	4E-34–3E-16	8–25			
